# Shape modes of $\mathbb {C}P^1$ vortices

**DOI:** 10.1007/s11005-026-02156-7

**Published:** 2026-09-29

**Authors:** Nora Gavrea, Derek Harland, Martin Speight

**Affiliations:** School of Mathematics, University of Leeds, Leeds, LS2 9JT UK

**Keywords:** Topological solitons, Vortices, Gauge theory, BPS equations, Shape modes, 81T13, 70S15, 35J10, 35C08, 58E15

## Abstract

In this paper, we investigate the existence of internal modes of vortices in the gauged $\mathbb {C}P^1$ sigma model. We develop a clean geometric formalism that highlights the symmetries of the Jacobi operator, obtained from the second variation of the energy functional. The formalism and subsequent results fundamentally rely on the Bogomol’nyi decomposition of the energy functional and can therefore be extended to other models with such a decomposition. We prove the existence of at least one shape mode for a general $\mathbb {C}P^1$ vortex solution on $\mathbb {R}^2$ and find numerically the shape modes and corresponding frequencies of a radially symmetric vortex. A surprising result is that the shape mode eigenvalues are very close to the scattering threshold, suggesting weakly bound shape modes could be characteristic of the $\mathbb {C}P^1$ model.

## Introduction

The gauged $\mathbb {C}P^1$ sigma model (also known as the gauged *O*(3) sigma model) was originally developed by Schroers [24]. It breaks the scale invariance of the pure $\mathbb {C}P^1$ sigma model by gauging a *U*(1) symmetry. After including a suitable potential term, the model admits soliton solutions which provide minima of the associated energy functional within their homotopy class. They are conventionally called BPS as they arise as solutions of a first-order PDE system obtained from a Bogomol’nyi-type argument [23, 24]. This model and the abelian Higgs model have some similarities, for example the *U*(1) symmetry of the fields and the fact that their solutions carry a quantized magnetic flux which makes them topologically stable. Solitons in both models are referred to as vortices.

In the context of the abelian Higgs model, vortices have been studied from different perspectives and have become relevant in the study of both cosmology and superconductivity. In cosmology, they describe large-scale cosmic strings which lead to structure formation due to local concentrations of energy [29]. In the Ginzburg-Landau theory of superconductivity, they describe magnetic flux tubes occurring in a type-II superconducting material after it is cooled down below its critical temperature and a magnetic field is applied [1]. Similar applications have been discovered for the $\mathbb {C}P^1$ vortices. In [34], Yang has shown how to construct $\mathbb {C}P^1$ vortices and anti-vortices coupled to gravity, representing cosmic strings and anti-strings with opposite magnetic charges. The $\mathbb {C}P^1$ model has also been proposed to model the competition between superconducting phases, due to its target space $\mathbb {C}P^1\cong S^2$ which comes with an additional constraint, allowing competition between a superconducting component and a charge density wave component [16, 27].

The gauged $\mathbb {C}P^1$ model has two interesting new features with no counterpart in the abelian Higgs model. First it carries an extra parameter $\tau \in (-1,1)$, the height of the vacuum manifold on $S^2$. (The analogous parameter in the abelian Higgs model can be scaled away.) This parameter affects the size and energy of vortices nontrivially. Second, due to its compact target space, the $\mathbb {C}P^1$ model exhibits two different types of vortices. Both these features were first noticed by Kimm, Lee and Lee [17], in the context of the model with a Chern-Simons term and additional neutral scalar field. In general, vortex positions are the points in physical space Σ that are mapped by the Higgs field ϕ to a fixed point of the action of the gauge group *U*(1) on the target space. In the abelian Higgs model, the target space $\mathbb {C}$ has only one fixed point, the origin, so there is only one species of vortex. In the gauged $\mathbb {C}P^1$ model, the target space $S^2$ has two fixed points which we may take, without loss of generality, to be the $\pm \boldsymbol{n}=(0,0,\pm 1)$, hence two distinct species of vortex. These may generically coexist. Finite energy field configurations carry a pair of integer valued topological invariants, the numbers $k_+$, $k_-$ of preimages of these fixed points counted with orientation and multiplicity. Every static energy minimizer in the $(k_+,k_-)$ class defines a disjoint pair of effective divisors on Σ of degrees $k_+$ and $k_-$ consisting of the preimages $D_+=\phi ^{-1}(\boldsymbol{n})$ and $D_-=\phi ^{-1}(-\boldsymbol{n})$. $D_+\cap D_-=\emptyset$ since no point can be mapped simultaneously to both $\boldsymbol{n}$ and $-\boldsymbol{n}$. Conversely, given a disjoint pair of effective divisors $D_+,D_-$ of degrees $(k_+,k_-)$ there is a static vortex in the degree $(k_+,k_-)$ class, unique up to gauge, with $\phi ^{-1}(\pm \boldsymbol{n})=D_\pm$. This was proved on $\Sigma =\mathbb {R}^2$ by Yang in the symmetric case τ=0 [33] and Han for general τ in [15]. On a compact Riemann surface Σ (of sufficiently large area), this was proved by Sibner, Sibner and Yang in the special case τ=0 in [25] and in the case of general τ by Garcia Lara [14]. The space of all these $(k_+,k_-)$ vortex solutions modulo gauge equivalence is called the moduli space, which is a generically non-compact complex manifold of dimension $k_++k_-$. The moduli space has a natural Riemannian metric whose geodesic flow models the dynamics of slowly moving vortices. A detailed analysis of the geometry of the moduli space of $\mathbb {C}P^1$ vortices is given in [23].

Our focus in this paper is the perturbative modes around a BPS vortex solution, along with the frequency spectrum of these modes. They solve an eigenvalue problem of a certain elliptic operator, arising from the second variation of the energy functional of the model. Quantum mechanically, they can be interpreted as particle states: they can be either bound states (particles trapped in the core of the vortex) or unbound states (particles scattering with the vortex). Bound states have $L^2$ normalizable eigenfunctions, while scattering states do not. By a shape mode, we will mean a bound state with strictly positive eigenvalue. (Bound modes with eigenvalue 0 correspond to perturbations tangent to the space of static solutions; all such modes are generated by moving the vortex positions, or by gauge transformations.)

In classical dynamics, shape modes correspond to spatially localized oscillatory motions of the time-dependent field equations linearized about the static vortex. Their existence, frequency and degeneracy are known to have profound effects on the low energy scattering of abelian Higgs vortices [3, 4, 8, 18, 22], and they have been intensively investigated in that model [5, 6, 10] by numerical means. But to date there are no rigorous analytic existence results for shape modes, either in the abelian Higgs model or the gauged $\mathbb {C}P^1$ model.

In this paper, we investigate the existence of shape modes of vortices in the $\mathbb {C}P^1$ model, focusing on the case $\Sigma =\mathbb {R}^2$. Rather than following the supersymmetry argument developed in [6] for radially symmetric abelian Higgs vortices, we develop a new formalism which allows for a cleaner treatment of the second variation of the energy functional as well as the eigenvalue problem giving rise to the internal modes, working initially on a general surface Σ, and later specializing to $\mathbb {R}^2$. This formalism fundamentally exploits the Bogomol’nyi energy decomposition; hence, in principle, it can be applied to any model with this property. It can be easily applied to vortices in the abelian Higgs model with slight modifications, and it could also be applied to other types of topological solitons (such as monopoles or lumps). We present this formalism and its implications in Sect. 3, where we prove the existence of at least one shape mode for a general $\mathbb {C}P^1$ vortex solution on $\Sigma =\mathbb {R}^2$, which is the main result of this paper. In Sect. 4, we focus on the radially symmetric case with vortices coincident at the origin and use our previous results to compute the shape modes numerically. We find that for a charge one vortex, the shape mode eigenvalue is very close to the scattering threshold, suggesting that bound states in the $\mathbb {C}P^1$ model are only weakly bound.

## The gauged $\mathbb {C}{\text {P}}^1$ sigma model

### The energy functional

Let Σ be an oriented Riemannian surface, and $P\rightarrow \Sigma$ a principal U(1)-bundle equipped with a connexion A. Let $\boldsymbol{\phi }$ be a section of the associated bundle $P\times _{\rho }M\rightarrow \Sigma$, where M is the target manifold, and ρ is a Hamiltonian action of U(1) on M.

In this paper, we consider $\Sigma =\mathbb {R}^2$ and $M=\mathbb {C}P^1$ which we identify as $S^2$ with the round metric. The action of U(1) on $M=S^2$ is represented by rotations about the z-axis. We will often adhere to the notation Σ, before we specialize our results to the particular case $\Sigma =\mathbb {R}^2$. In this later case, P is trivial and the Higgs field is a function $\boldsymbol{\phi }:\mathbb {R}^2\rightarrow S^2$.

We are interested in finding pairs $(A,\boldsymbol{\phi })$ which are global minimizers of the energy functional

2.1 $$\begin{aligned} E[\boldsymbol{\phi }, A]=\dfrac{1}{2}\int _{\Sigma } \left[ \left| F\right| ^2+\left| D\boldsymbol{\phi }\right| ^2+\left| \tau -\boldsymbol{n}\cdot \boldsymbol{\phi }\right| ^2\right] , \end{aligned}$$

where $\boldsymbol{n}=(0,0,1)$ is the constant unit vector pointing in the z-direction, F=dA is the curvature of the connexion, and $D\boldsymbol{\phi }=d\boldsymbol{\phi }-A\boldsymbol{n}\times \boldsymbol{\phi }$ is the covariant derivative in a local trivialization of $P\times _\rho M$. The parameter $\tau \in (-1,1)$ in the potential $U(\boldsymbol{\phi })=(\tau -\boldsymbol{n}\cdot \boldsymbol{\phi })^2$ defines the vacuum manifold, where the potential attains its minimum value equal to zero. (The case $\tau =\pm 1$ is somewhat pathological, and will not be considered here.)

For the non-compact case $\Sigma =\mathbb {R}^2$, finite energy solutions require the following boundary conditions:

2.2 $$\begin{aligned} \lim _{|\boldsymbol{x}|\rightarrow \infty }F=0, \lim _{|\boldsymbol{x}|\rightarrow \infty }D\boldsymbol{\phi }=0, \lim _{|\boldsymbol{x}|\rightarrow \infty }\boldsymbol{n}\cdot \boldsymbol{\phi }=\tau . \end{aligned}$$

### Gauge transformations

The energy functional (2.1) is invariant under the following local gauge transformations

$$ \boldsymbol{\phi }\mapsto R(\theta )\boldsymbol{\phi } $$

$$ A\mapsto A+d\theta , $$

where $\theta :\Sigma \rightarrow \mathbb {R}$ and $R(\theta )\in SO(3)$ represents the rotation with angle θ about the z-axis

$$ R(\theta )=\begin{pmatrix} \cos {\theta } &  -\sin {\theta } &  0\\ \sin {\theta } &  \cos {\theta } &  0 \\ 0 &  0 &  1 \end{pmatrix}. $$

### The Bogomol’nyi equations

The energy functional admits a Bogomol’nyi-type decomposition, which results from completing the square inside the energy functional:

2.3 $$\begin{aligned} E[\boldsymbol{\phi }, A]=\dfrac{1}{2}\int _{\Sigma } \left[ \dfrac{1}{2}\left| D\boldsymbol{\phi }- *\boldsymbol{\phi }\times D\boldsymbol{\phi }\right| ^2+\left| *F+ (\tau -\boldsymbol{n}\cdot \boldsymbol{\phi })\right| ^2\right] \end{aligned}$$

$$ +\int _{\Sigma } \left[ \dfrac{1}{2}D\boldsymbol{\phi }\cdot \boldsymbol{\phi }\times D\boldsymbol{\phi }- (\tau -\boldsymbol{n}\cdot \boldsymbol{\phi })F\right] . $$

It can be shown [23] that the cross term integrates to

$$ \int _{\Sigma } \left[ \dfrac{1}{2}D\boldsymbol{\phi }\cdot \boldsymbol{\phi }\times D\boldsymbol{\phi }- (\tau -\boldsymbol{n}\cdot \boldsymbol{\phi })F\right] =2\pi (1-\tau )k_++2\pi (1+\tau )k_-, $$

under the assumption that F, $D\boldsymbol{\phi }$ and $\boldsymbol{n}\cdot \boldsymbol{\phi }$ decay sufficiently rapidly on the boundary of Σ.

The numbers $k_+$ and $k_-$ are integers encoding the number of North vortices and South anti-vortices on Σ, respectively: the pre-image of the North pole under $\boldsymbol{\phi }$ is a set of $k_+$ points on Σ, representing the locations of the North vortices, and the pre-image of the South pole under $\boldsymbol{\phi }$ is a set of $k_-$ points on Σ, representing the locations of the South anti-vortices. These (anti)-vortices are topologically distinct, and their coexistence was established by Yang [34]. The difference between vortices and anti-vortices arises from the fact that local coordinate systems in the neighborhoods of the North and South poles have opposite orientations. Thus, the field winds around the vacuum manifold $k_+$ times in anticlockwise direction, corresponding to the contribution of the North vortices, and $k_-$ times in clockwise direction, corresponding to the contribution of the South anti-vortices. These windings are also related to the total magnetic flux through the surface [27], which is quantized and can be computed

$$ \dfrac{1}{2\pi }\int _{\Sigma }F=k_+-k_-. $$

From the above Bogomol’nyi argument, we conclude the energy is bounded below by the topological invariant given by the integration of the cross terms, and the inequality is saturated when the first-order Bogomol’nyi equations hold

2.4 $$\begin{aligned} D\boldsymbol{\phi }=*\boldsymbol{\phi }\times D\boldsymbol{\phi } \end{aligned}$$

2.5 $$\begin{aligned} *F=-(\tau -\boldsymbol{n}\cdot \boldsymbol{\phi }), \end{aligned}$$

with solutions $(A,\boldsymbol{\phi })$ being conventionally called BPS solutions. It has been shown that, up to gauge equivalence, BPS configurations are determined by the pair of divisors $(D_+,D_-)$ with degrees $(k_+,k_-)$ on Σ, specifying the locations of the vortices and anti-vortices, respectively, including their algebraic multiplicities [23, 33].

## Shape modes of a $\mathbb {C}{\text {P}}^1$ vortex solution

Recall the shape modes represent the perturbative modes around a BPS vortex solution. They are eigenmodes of the second-order elliptic operator obtained from the second variation of the energy functional (2.1), with positive eigenvalue. They offer extensive information about the dynamics of excited vortices near the critical BPS point in the moduli space. Such dynamics is rich and complicated, and has been widely studied recently in the context of the Abelian-Higgs model [4, 7, 8, 18], revealing surprising phenomena, such as for example the existence of spectral walls [9].

To investigate shape modes in the context of the $\mathbb {C}P^1$ model, we must first find the second variation of the energy functional (2.1) around a BPS solution $(\boldsymbol{\phi },A)$. It is helpful to work initially on a general surface Σ, and later set $\Sigma =\mathbb {R}^2$. Throughout we assume that P is trivial. We will start by computing the first variation and defining some new operators, as this establishes a good starting point for the second variation whose calculation is more computationally difficult.

### The first variation

Let $(\boldsymbol{\phi }_s,A_s)$ be a 1-parameter variation of the BPS solution $(\boldsymbol{\phi }_0,A_0)=(\boldsymbol{\phi },A)$. This generates the infinitesimal perturbation

$$ \dfrac{d}{ds}\Bigg \vert _{s=0}\boldsymbol{\phi }_s(\boldsymbol{x})=\boldsymbol{\eta }(\boldsymbol{x}), \dfrac{d}{ds}\Bigg \vert _{s=0}A_s(\boldsymbol{x})=\alpha (\boldsymbol{x}). $$

We will consider the energy functional written in Bogomol’nyi form, which will later enable us to write the second variation operator as a composition of two first-order operators. This approach becomes very helpful once we try to prove existence of and construct the shape modes. We have the energy

$$ E[\boldsymbol{\phi }, A]=\dfrac{1}{4}\Vert D\boldsymbol{\phi }-*\boldsymbol{\phi }\times D\boldsymbol{\phi }\Vert _{L^2}^2+\dfrac{1}{2}\Vert *F+(\tau -\boldsymbol{n}\cdot \boldsymbol{\phi })\Vert ^2_{L^2}+E_0, $$

where $E_0=2\pi (1-\tau )k_++2\pi (1+\tau )k_-$, and the $L^2$ inner product is given by

$$ \left\langle \alpha ,\beta \right\rangle _{L^2}=\int _{\Sigma } \alpha \wedge *\beta . $$

By defining the operator

$$\begin{aligned}Bog: C^\infty \left( \Sigma , S^2\right) \oplus \Gamma {(T^*\Sigma )}&\rightarrow \Gamma {(T^*\Sigma \otimes \boldsymbol{\phi }^*TS^2)}\oplus C^\infty (\Sigma ) \\ \begin{pmatrix} \boldsymbol{\phi } \\ A \end{pmatrix}&\mapsto \begin{pmatrix} \dfrac{1}{\sqrt{2}}\left( D\boldsymbol{\phi }-*\boldsymbol{\phi }\times D\boldsymbol{\phi }\right) \\ *F+(\tau -\boldsymbol{n}\cdot \boldsymbol{\phi }) \end{pmatrix}, \end{aligned}$$

we can rewrite the energy more compactly as

3.1 $$\begin{aligned} E[\boldsymbol{\phi }, A]=\dfrac{1}{2}\left\langle Bog(\boldsymbol{\phi },A), Bog(\boldsymbol{\phi }, A)\right\rangle _{L^2}+E_0. \end{aligned}$$

Then the first variation of the energy is given by

$$ \dfrac{d}{ds}\Bigg |_{s=0}E[\boldsymbol{\phi }_s,A_s]=\left\langle \dfrac{d}{ds}\Bigg |_{s=0}Bog(\boldsymbol{\phi }_s,A_s), Bog(\boldsymbol{\phi }_s, A_s)\right\rangle _{L^2}=\left\langle \mathscr {B}\boldsymbol{\xi }, Bog(\boldsymbol{\phi }, A)\right\rangle _{L^2}, $$

where $$\boldsymbol{\xi }=\begin{pmatrix} \boldsymbol{\eta }\\ \alpha \end{pmatrix}$$ and $\mathscr {B}$ is the differential of the map *Bog* at (ϕ,A):

$$\begin{aligned}\mathscr {B}:\Gamma (\boldsymbol{\phi }^*TS^2)\oplus \Gamma (T^{*}\Sigma )&\rightarrow \Gamma (T^{*}\Sigma \otimes \boldsymbol{\phi }^*TS^2)\oplus C^{\infty }(\Sigma )\\ \boldsymbol{\xi }=\begin{pmatrix}\boldsymbol{\eta }\\ \alpha \end{pmatrix}&\mapsto \begin{pmatrix} \dfrac{1}{\sqrt{2}}\left( D\boldsymbol{\eta }-\alpha \boldsymbol{n}\times \boldsymbol{\phi }-*\boldsymbol{\eta }\times D\boldsymbol{\phi }-*\boldsymbol{\phi }\times (D\boldsymbol{\eta }-\alpha \boldsymbol{n}\times \boldsymbol{\phi })\right) \\ *d\alpha -\boldsymbol{n}\cdot \boldsymbol{\eta } \end{pmatrix}. \end{aligned}$$

The domain of this operator will occur frequently in the sequel, so it is useful to give it a name. Since it is the space of linear perturbations about the static vortex $(\boldsymbol{\phi },A)$, we call it

3.2 $$\begin{aligned} \mathcal {PERT}:=\Gamma (\boldsymbol{\phi }^*TS^2)\oplus \Gamma (T^*\Sigma ) \end{aligned}$$

To rewrite the operator $\mathscr {B}$ more compactly and to ensure an easier calculation for the second variation in the next subsection, it is helpful to define the following linear operators

3.3 $$\begin{aligned} L_{\boldsymbol{\phi }}: \Gamma (T^{*}\Sigma \otimes \boldsymbol{\phi }^{*} TS^2)\rightarrow &   \Gamma (T^{*}\Sigma \otimes \boldsymbol{\phi }^{*} TS^2)\nonumber \\ \boldsymbol{\eta }\mapsto &   \frac{1}{\sqrt{2}}\left( \boldsymbol{\eta }-\boldsymbol{\phi }\times *\boldsymbol{\eta }\right) \end{aligned}$$

3.4 $$\begin{aligned} P_{\boldsymbol{\phi }}:\Gamma (T^{*}\Sigma \otimes \mathbb {R}^3)\rightarrow &   \Gamma (T^{*}\Sigma \otimes \boldsymbol{\phi }^{*} TS^2)\nonumber \\ \boldsymbol{u}\mapsto &   \boldsymbol{u}-(\boldsymbol{u}\cdot \boldsymbol{\phi })\boldsymbol{\phi }. \end{aligned}$$

One easily verifies that

3.5 $$\begin{aligned} L_{\boldsymbol{\phi }}\circ L_{\boldsymbol{\phi }}&=\sqrt{2}L_{\boldsymbol{\phi }} \end{aligned}$$

3.6 $$\begin{aligned} P_{\boldsymbol{\phi }}\circ P_{\boldsymbol{\phi }}&=P_{\boldsymbol{\phi }} \end{aligned}$$

3.7 $$\begin{aligned} \left[ L_{\boldsymbol{\phi }},*\right]&=0 \end{aligned}$$

3.8 $$\begin{aligned} \left[ L_{\boldsymbol{\phi }},P_{\boldsymbol{\phi }}\right]&=0 \end{aligned}$$

3.9 $$\begin{aligned} L_{\boldsymbol{\phi }}^{\dagger }&=L_{\boldsymbol{\phi }} \end{aligned}$$

3.10 $$\begin{aligned} P_{\boldsymbol{\phi }}^{\dagger }&=P_{\boldsymbol{\phi }}, \end{aligned}$$

where the “dagger" superscript denotes the $L^2$-adjoint of the respective operator, and

3.11 $$\begin{aligned} \mathscr {B}\begin{pmatrix}\boldsymbol{\eta }\\ \alpha \end{pmatrix}= \begin{pmatrix} L_{\boldsymbol{\phi }}\circ P_{\boldsymbol{\phi }}\left( D\boldsymbol{\eta }-\alpha \boldsymbol{n}\times \boldsymbol{\phi }\right) \\ *d\alpha -\boldsymbol{n}\cdot \boldsymbol{\eta } \end{pmatrix}. \end{aligned}$$

### The second variation

Let $(\boldsymbol{\phi }_{s,t},A_{s,t})$ be a 2-parameter variation of the BPS solution $(\boldsymbol{\phi }_{0,0},A_{0,0})=(\boldsymbol{\phi },A)$. This generates the infinitesimal perturbations

$$ \dfrac{\partial }{\partial s}\Bigg \vert _{s=0}\boldsymbol{\phi }_{s,t}(\boldsymbol{x})=\boldsymbol{\eta }_t(\boldsymbol{x}), \dfrac{\partial }{\partial s}\Bigg \vert _{s=0}A_{s,t}(\boldsymbol{x})=\alpha _t(\boldsymbol{x}) $$

$$ \dfrac{\partial }{\partial t}\Bigg \vert _{s=t=0}\boldsymbol{\phi }_{s,t}(\boldsymbol{x})=\tilde{\boldsymbol{\eta }}(\boldsymbol{x}), \dfrac{\partial }{\partial t}\Bigg \vert _{s=t=0}A_{s,t}(\boldsymbol{x})=\tilde{\alpha }(\boldsymbol{x}). $$

Let $$\boldsymbol{\xi }=(\boldsymbol{\eta }_0,\alpha _0)$$ and $$\tilde{\boldsymbol{\xi }}=(\tilde{\boldsymbol{\eta }},\tilde{\alpha })$$

The second variation of the energy functional around a BPS solution is then given by

3.12 $$\begin{aligned} \dfrac{\partial }{\partial t}\Bigg \vert _{t=0}\dfrac{\partial }{\partial s}\Bigg \vert _{s=0}E[\boldsymbol{\phi }_{s,t}, A_{s,t}]= &   \left\langle \dfrac{\partial }{\partial s}\Bigg |_{s=0}Bog(\boldsymbol{\phi }_{s,t},A_{s,t}), \dfrac{\partial }{\partial t}\Bigg |_{t=0}Bog(\boldsymbol{\phi }_{s,t}, A_{s,t})\right\rangle _{L^2}\nonumber \\= &   \left\langle \mathscr {B}\boldsymbol{\xi }, \mathscr {B}\tilde{\boldsymbol{\xi }}\right\rangle _{L^2}\nonumber \\= &   \left\langle \boldsymbol{\xi }, \mathscr {B}^{\dagger }\mathscr {B}\tilde{\boldsymbol{\xi }}\right\rangle _{L^2}, \end{aligned}$$

where

3.13 $$\begin{aligned} \begin{aligned}\mathscr {B}^{\dagger }: \Gamma (T^{*}\Sigma \otimes \boldsymbol{\phi }^*TS^2)\oplus C^{\infty }(\Sigma )&\rightarrow \mathcal {PERT}\\ \begin{pmatrix}\boldsymbol{\chi _1}\\ \chi _2\end{pmatrix}&\mapsto \begin{pmatrix} P_{\boldsymbol{\phi }}\left( -*D*L_{\boldsymbol{\phi }}(\boldsymbol{\chi _1})-\boldsymbol{n}\chi _2\right) \\ -*d\chi _2-(\boldsymbol{n}\times \boldsymbol{\phi })\cdot L_{\boldsymbol{\phi }}\left( \boldsymbol{\chi }_1\right) \end{pmatrix} \end{aligned} \end{aligned}$$

is the $L^2$-adjoint of $\mathscr {B}$ expressed in (3.11). Its calculation follows quickly by using the properties listed in (3.5–3.10).

Recall that the perturbation $\boldsymbol{\eta }$ comes with the constraint $\boldsymbol{\eta }(\boldsymbol{x})\in T_{\boldsymbol{\phi }(\boldsymbol{x})}S^2$, and hence, we should be careful with handling inner products of the type $\left\langle \boldsymbol{\eta }, \cdot \right\rangle _{L^2}$ when deriving expression (3.13), since only the terms perpendicular to $\boldsymbol{\phi }$ bring a contribution, as the ones parallel to $\boldsymbol{\phi }$ equate to zero when taken in the inner product with $\boldsymbol{\eta }$. To rule out the occurence of such extraneous terms, we note $\left\langle \boldsymbol{\eta }, \cdot \right\rangle _{L^2}=\left\langle \boldsymbol{\eta },P_{\boldsymbol{\phi }}(\cdot )\right\rangle _{L^2}$.

Note from (3.12) we can now identify the second variation operator $\mathscr {J}$, called the Jacobi operator, with $\mathscr {J}=\mathscr {B}^{\dagger }\mathscr {B}$. This decomposition reinforces more obviously that $\mathscr {J}$ is a positive semidefinite self-adjoint operator.

An important observation is that we were able to decompose the Jacobi operator into a product of two first-order operators, one of which is the $L^2$-adjoint of the other, simply due to the fact that the energy functional admits a Bogomol’nyi argument. Hence, this represents a general property of this type of models.

### Zero modes and the orthogonal gauge condition

Recall the energy functional (2.1) is invariant under the gauge transformations presented in Sect. 2.2. Therefore, perturbations of the fields represented by infinitesimal gauge transformations leave the Bogomol’nyi equations invariant, and lie in the kernel of the operator $\mathscr {B}$.

Let us denote by $\mathscr {G}$ the map which assigns to a smooth real function on Σ the infinitesimal gauge transformation that it generates:

3.14 $$\begin{aligned} \mathscr {G}:C^\infty (\Sigma )\rightarrow &   \mathcal {PERT}\nonumber \\ \chi\mapsto &   (\chi \boldsymbol{n}\times \boldsymbol{\phi },d\chi ). \end{aligned}$$

As we have just observed, $\mathscr {B}\mathscr {G}=0$, that is, infinitesimal gauge transformations lie in the kernel of $\mathscr {B}$. If we wish to contruct the tangent space to the vortex moduli space at (the gauge equivalence class of) $(\boldsymbol{\phi },A)$, we should keep only those vectors in $\ker \mathscr {B}$ which are $L^2$ orthogonal to all such gauge transformations, i.e., $\langle \mathscr {G}(\chi ),(\boldsymbol{\eta },\alpha )\rangle _{L^2}=0$ for all χ. Clearly this is equivalent to

3.15 $$\begin{aligned} \mathscr {G}^\dagger (\boldsymbol{\eta },\alpha )=0 \end{aligned}$$

where

3.16 $$\begin{aligned} \mathscr {G}^\dagger :\mathcal {PERT}\rightarrow &   C^\infty (\Sigma )\nonumber \\ (\boldsymbol{\eta },\alpha )\mapsto &   -*d *\alpha +\boldsymbol{\eta }\cdot \left( \boldsymbol{n}\times \boldsymbol{\phi }\right) \end{aligned}$$

is the $L^2$ adjoint of $\mathscr {G}$.

Hence, $T_{[(\boldsymbol{\phi },A)]}\textsf{M}$, the tangent space of the moduli space $\textsf{M}$ at the point $[(\boldsymbol{\phi },A)]$, is precisely the kernel of the extended operator

3.17 $$\begin{aligned} \mathscr {B}^G:=\mathscr {B}\oplus \mathscr {G}^\dagger : \mathcal {PERT}\rightarrow \mathcal {BOG} \end{aligned}$$

whose codomain we have denoted

$$ \mathcal {BOG}:=\Gamma (T^{*}\Sigma \otimes \boldsymbol{\phi }^*TS^2)\oplus C^{\infty }(\Sigma )\oplus C^{\infty }(\Sigma ). $$

The $L^2$-adjoint of $\mathscr {B}^G$ is

3.18 $$\begin{aligned} \begin{aligned} \mathscr {B}^{G\dagger }: \mathcal {BOG}&\rightarrow \mathcal {PERT}\\ \begin{pmatrix} \boldsymbol{\chi }_1 \\ \chi _2\\ \chi _3 \end{pmatrix}&\mapsto \begin{pmatrix} P_{\boldsymbol{\phi }}\left( -*D*L_{\boldsymbol{\phi }}(\boldsymbol{\chi _1})-\boldsymbol{n}\chi _2\right) +\boldsymbol{n}\times \boldsymbol{\phi }\chi _3\\ -*d\chi _2-(\boldsymbol{n}\times \boldsymbol{\phi })\cdot L_{\boldsymbol{\phi }}\left( \boldsymbol{\chi }_1\right) +d\chi _3 \end{pmatrix}, \end{aligned} \end{aligned}$$

using the properties (3.5–3.10).

Since $\mathscr {J}=\mathscr {B}^\dagger \mathscr {B}$, it follows immediately that

3.19 $$\begin{aligned} \mathscr {J}\mathscr {G}=0,\qquad \mathscr {G}^\dagger \mathscr {J}=0. \end{aligned}$$

Hence, every eigenmode of $\mathscr {J}$ not in its kernel automatically satisfies the gauge orthogonality condition (3.15).

### The extended Jacobi operator

We can now define the extended Jacobi operator by

3.20 $$\begin{aligned} \mathscr {J}^G:=\mathscr {B}^{G\dagger }\mathscr {B}^G=\mathscr {B}^{\dagger }\mathscr {B}+\mathscr {G}\mathscr {G}^{\dagger }=\mathscr {J}+\mathscr {G}\mathscr {G}^{\dagger }:\mathcal {PERT}\rightarrow \mathcal {PERT}. \end{aligned}$$

We will prove shortly that $\mathscr {J}$ and $\mathscr {J}^G$ have the same eigenvalue spectrum. A formula for this operator can be computed by putting together (3.17) and (3.18), giving

$$ \mathscr {J}^G \begin{pmatrix} \boldsymbol{\eta } \\ \alpha \end{pmatrix}=\begin{pmatrix} -*\hat{D}*\hat{D}\boldsymbol{\eta }-\boldsymbol{\phi }\times *\hat{D}^2\boldsymbol{\eta }-2(\boldsymbol{n}\cdot \boldsymbol{\phi })*(D\boldsymbol{\phi }\wedge \alpha )+|\boldsymbol{n}\times \boldsymbol{\phi }|^2\boldsymbol{\eta }\\ \Delta \alpha -2(\boldsymbol{n}\cdot \boldsymbol{\phi })(\boldsymbol{\eta }\cdot *D\boldsymbol{\phi })+\alpha |\boldsymbol{n}\times \boldsymbol{\phi }|^2 \end{pmatrix}, $$

where $\hat{D}=P_{\boldsymbol{\phi }}\circ D$, and Δ is the Hodge Laplacian.

Analyzing the structure of the operators $\mathscr {B}^G$ and $\mathscr {B}^{G\dagger }$, it is helpful to define the $L^2$ isometries

3.21 $$\begin{aligned} \begin{aligned} \mathscr {S}_1:\mathcal {PERT}&\rightarrow \mathcal {PERT} \\ \begin{pmatrix} \boldsymbol{\eta }\\ \alpha \end{pmatrix}&\mapsto \begin{pmatrix} \boldsymbol{\phi }\times \boldsymbol{\eta }\\ *\alpha \end{pmatrix} \end{aligned} \end{aligned}$$

3.22 $$\begin{aligned} \begin{aligned} \mathscr {S}_2:\mathcal {BOG}&\rightarrow \mathcal {BOG} \\ \begin{pmatrix} \boldsymbol{\chi }_1\\ \chi _2\\ \chi _3 \end{pmatrix}&\mapsto \begin{pmatrix} *\boldsymbol{\chi }_1\\ -\chi _3\\ \chi _2 \end{pmatrix}. \end{aligned} \end{aligned}$$

The following lemma concerns the properties of the maps (3.21) and (3.22) and the symmetries of the operators $\mathscr {J}^G$ and $\mathscr {B}^{G}\mathscr {B}^{G\dagger }$.

#### Lemma 3.1

The maps $\mathscr {S}_1$ and $\mathscr {S}_2$ satisfy the following properties

3.23 $$\begin{aligned} \mathscr {S}_1\circ \mathscr {S}_1&=-id_{\mathcal {PERT}} \end{aligned}$$

3.24 $$\begin{aligned} \mathscr {S}_2\circ \mathscr {S}_2&=-id_{\mathcal {BOG}}\end{aligned}$$

3.25 $$\begin{aligned} \mathscr {S}_1^{\dagger }&=-\mathscr {S}_1\end{aligned}$$

3.26 $$\begin{aligned} \mathscr {S}_2^{\dagger }&=-\mathscr {S}_2\end{aligned}$$

3.27 $$\begin{aligned} \mathscr {G}^{\dagger }\mathscr {S}_1\mathscr {G}&=0 \end{aligned}$$

Furthermore, $\left[ \mathscr {J}^G,\mathscr {S}_1\right] =0$ and $\left[ \mathscr {B}^G\mathscr {B}^{G\dagger },\mathscr {S}_2\right] =0$, i.e., they represent symmetries of the operators $\mathscr {J}^G$ and $\mathscr {B}^{G}\mathscr {B}^{G\dagger }$, respectively.

#### Proof

From the definitions of the two maps given in (3.21) and (3.22), it is immediate to check that the properties (3.23–3.26) hold.

To prove (3.27), we compute directly by using the definitions (3.14), (3.16), (3.21)

$$ \mathscr {G}^{\dagger }\mathscr {S}_1\mathscr {G}\chi =\mathscr {G}^{\dagger }\begin{pmatrix} \boldsymbol{\phi }\times (\boldsymbol{n}\times \boldsymbol{\phi })\chi \\ *d\chi \end{pmatrix}=-*d*\left( *d\chi \right) +\left[ \boldsymbol{\phi }\times (\boldsymbol{n}\times \boldsymbol{\phi })\chi \right] \cdot (\boldsymbol{n}\times \boldsymbol{\phi })=0. $$

From the expressions (3.17) and (3.18), we can easily check that

3.28 $$\begin{aligned} \mathscr {B}^G\mathscr {S}_1=\mathscr {S}_2\mathscr {B}^G \end{aligned}$$

3.29 $$\begin{aligned} \mathscr {B}^{G\dagger }\mathscr {S}_2=\mathscr {S}_1\mathscr {B}^{G\dagger }, \end{aligned}$$

where the second equation can also be obtained by taking the $L^2$-adjoint of the first one.

Applying $\mathscr {B}^{G\dagger }$ to (3.28), we obtain

$$ \mathscr {B}^{G\dagger }\mathscr {B}^G\mathscr {S}_1=\mathscr {B}^{G\dagger }\mathscr {S}_2\mathscr {B}^G=\mathscr {S}_1\mathscr {B}^{G\dagger }\mathscr {B}^G, $$

where in the second equality we used (3.29). Hence, the operators $\mathscr {J}^G$ and $\mathscr {S}_1$ commute

3.30 $$\begin{aligned} \mathscr {J}^G\mathscr {S}_1=\mathscr {S}_1\mathscr {J}^G, \end{aligned}$$

which is equivalent to saying that $\mathscr {S}_1$ is a symmetry of the operator $\mathscr {J}^G$. Similarly, applying $\mathscr {B}^G$ to (3.29) yields

3.31 $$\begin{aligned} \mathscr {B}^{G}\mathscr {B}^{G\dagger }\mathscr {S}_2=\mathscr {S}_2\mathscr {B}^{G}\mathscr {B}^{G\dagger }. \end{aligned}$$

□

#### Remarks

1. The map $\mathscr {S}_1$ is an isometry of $\mathcal {PERT}$ (with respect to its natural $L^2$ inner product) which squares to -id. Hence, it may naturally be interpreted as an almost complex structure on $\mathcal {PERT}$. Its restriction to $\ker \mathscr {B}^G$ is precisely the natural almost complex structure on the vortex moduli space.
2. The above Lemma implies that if $\boldsymbol{\xi }$ is an eigenmode of $\mathscr {J}^{G}$ with eigenvalue $\lambda ^2$, then so is $\mathscr {S}_1\boldsymbol{\xi }$, and similarly if $\boldsymbol{\chi }$ is an eigenmode of $\mathscr {B}^{G}\mathscr {B}^{G\dagger }$ with eigenvalue $\lambda ^2$, then so is $\mathscr {S}_2\boldsymbol{\chi }$. So eigenmodes of $\mathscr {J}^G$ come in degenerate pairs.
3. We also notice that if $\boldsymbol{\chi }$ is an eigenmode of $\mathscr {B}^{G}\mathscr {B}^{G\dagger }$ with eigenvalue $\lambda ^2$, then $\mathscr {B}^{G\dagger }\boldsymbol{\chi }$ (if non-zero) is an eigenmode of $\mathscr {J}^{G}$ with the same eigenvalue. This observation will turn out to be crucial later when we compute the shape modes, as the operator $\mathscr {B}^{G}\mathscr {B}^{G\dagger }$ allows for the decoupling of the PDE system obtained in the eigenvalue problem. This is because the operator can be written in block form. To see this, define
  3.32 $$\begin{aligned} \begin{aligned} \mathscr {T}:\mathcal {PERT}&\rightarrow \Gamma (T^{*}\Sigma \otimes \boldsymbol{\phi }^*TS^2) \\ \begin{pmatrix} \boldsymbol{\eta }\\ \alpha \end{pmatrix}&\mapsto L_{\boldsymbol{\phi }}\circ P_{\boldsymbol{\phi }}\left( D\boldsymbol{\eta }-\alpha \boldsymbol{n}\times \boldsymbol{\phi }\right) , \end{aligned} \end{aligned}$$
  where this operator represents the first component in (3.11), which immediately implies $\mathscr {T}\mathscr {G}=0$. Then notice we can rewrite (3.17) as
  $$ \mathscr {B}^G=\mathscr {T}\oplus \mathscr {G}^{\dagger }\mathscr {S}_1\oplus \mathscr {G}^{\dagger }. $$
  Then the $L^2$-adjoint is given by
  $$ \mathscr {B}^{G\dagger }\begin{pmatrix} \boldsymbol{\chi }_1 \\ \chi _2\\ \chi _3 \end{pmatrix}=\mathscr {T}^\dagger \boldsymbol{\chi }_1-\mathscr {S}_1\mathscr {G}\chi _2+\mathscr {G}\chi _3. $$
  A short calculation then shows that we can write the operator $\mathscr {B}^{G}\mathscr {B}^{G\dagger }$ in "block form":
  $$ \mathscr {B}^{G}\mathscr {B}^{G\dagger }=\begin{pmatrix} \mathscr {T}\mathscr {T}^\dagger &  -\mathscr {T}\mathscr {S}_1\mathscr {G} &  0\\ \mathscr {G}^\dagger \mathscr {S}_1\mathscr {T}^\dagger &  \mathscr {G}^\dagger \mathscr {G} &  0\\ 0 &  0 &  \mathscr {G}^\dagger \mathscr {G} \end{pmatrix}. $$
4. Finally, observe that if $\boldsymbol{\xi }\in \ker \mathscr {J}^G$, then
  $$ 0=\left\langle \boldsymbol{\xi },\mathscr {J}^G\boldsymbol{\xi }\right\rangle _{L^2}=\left\langle \mathscr {B}^{G}\boldsymbol{\xi },\mathscr {B}^{G}\boldsymbol{\xi }\right\rangle _{L^2}=\left| \mathscr {B}^{G}\boldsymbol{\xi }\right| ^2_{L^2}, $$
  implying $\mathscr {B}^{G}\boldsymbol{\xi }=0$. Hence, $\ker \mathscr {J}^G=\ker \mathscr {B}^G$, and similarly $\ker \mathscr {B}^{G}\mathscr {B}^{G\dagger }=\ker \mathscr {B}^{G\dagger }$.

Now that we are equipped with these new operators, we show that this formulation allows for an elegant and quick computation of a vortex shape mode.

#### Theorem 3.2

If $\psi :\Sigma \rightarrow \mathbb {R}$ is an $L^2$ eigenfunction of the Schrödinger operator

3.33 $$\begin{aligned} L=\Delta +|\boldsymbol{n}\times \boldsymbol{\phi }|^2 \end{aligned}$$

then $\mathscr {S}_1\mathscr {G}\psi$ is an $L^2$-integrable eigenmode of $\mathscr {J}$ with the same eigenvalue. Here Δ=-∗d∗d denotes the Laplace-Beltrami operator on Σ.

#### Proof

First observe that $L=\mathscr {G}^{\dagger }\mathscr {G}$. Applying $\mathscr {G}$ to the left, we observe that if $L\psi =\lambda ^2\psi$, then

$$ \mathscr {G}\mathscr {G}^\dagger (\mathscr {G}\psi )=\lambda ^2\mathscr {G}\psi $$

and $\ker \mathscr {G}=0$ by its definition (3.14), so $\mathscr {G}\psi$ is an eigenmode of $\mathscr {G}\mathscr {G}^{\dagger }$ with the same eigenvalue. Now $\mathscr {J}\mathscr {G}=0$ since, for all $\chi \in C^\infty (\Sigma )$, $\mathscr {G}(\chi )$ is an infinitesimal gauge transformation, all of which lie in the kernel of $\mathscr {J}$ by the gauge invariance of the energy functional. Hence,

$$ \mathscr {J}^G\mathscr {G}\psi =(\mathscr {J}+\mathscr {G}\mathscr {G}^{\dagger })\mathscr {G}\psi =\mathscr {G}\mathscr {G}^{\dagger }\mathscr {G}\psi =\lambda ^2\mathscr {G}\psi , $$

that is, $\mathscr {G}\psi$ is an eigenmode of $\mathscr {J}^G$ with eigenvalue $\lambda ^2$.

By the symmetry we proved in Lemma 3.1, we deduce that $\mathscr {S}_1\mathscr {G}\psi$ is also an eigenmode of $\mathscr {J}^G$. Then we have

$$ \mathscr {J}\mathscr {S}_1\mathscr {G}\psi =(\mathscr {J}^G-\mathscr {G}\mathscr {G}^\dagger )\mathscr {S}_1\mathscr {G}\psi = \mathscr {J}^G\left( \mathscr {S}_1\mathscr {G}\psi \right) =\lambda ^2\mathscr {S}_1\mathscr {G}\psi , $$

by (3.27) proved in Lemma 3.1. We therefore deduce that $\mathscr {S}_1\mathscr {G}\psi$ is also an eigenmode of $\mathscr {J}$.

The $L^2$-integrability part of the theorem follows quickly by noticing

$$ |\mathscr {S}_1\mathscr {G}\psi |^2_{L^2}=\left\langle \mathscr {S}_1\mathscr {G}\psi ,\mathscr {S}_1\mathscr {G}\psi \right\rangle _{L^2}=\left\langle \psi ,\mathscr {G}^{\dagger }\mathscr {S}_1^{\dagger }\mathscr {S}_1\mathscr {G}\psi \right\rangle _{L^2}=\lambda ^2\left\langle \psi ,\psi \right\rangle _{L^2}<\infty , $$

where we used (3.25), (3.23) and $\mathscr {G}^{\dagger }\mathscr {G}\psi =\lambda ^2\psi$. Hence, if ψ is $L^2$-integrable, then so is $\mathscr {S}_1\mathscr {G}\psi$. □

#### Remarks

1. Since the potential $|\boldsymbol{n}\times \boldsymbol{\phi }|^2$ in the Schrödinger operator is nonnegative and vanishes only on the divisors $D_\pm$, any $L^2$ eigenmode constructed in this way must have $\lambda ^2>0$, and so is a shape mode.
2. The above proposition reveals a powerful and quick method to construct a shape mode, by solving a single scalar PDE $L\psi =\lambda ^2\psi$. This is much simpler than solving the eigenvalue problem for $\mathscr {J}$ directly (a coupled system of 5 PDEs with a constraint). However, there is no guarantee that this method will give all possible shape modes.

To find all possible shape modes of a given vortex solution, we need to solve the eigenvalue problem for the operator $\mathscr {J}$, which fails to be elliptic. We next show that the eigenvalue spectrum of $\mathscr {J}$ coincides precisely with that of $\mathscr {J}^G$, which *is* elliptic. The proof will make use of the orthogonal decomposition

$$ \mathcal {PERT}=\mathcal {G}_\infty \oplus \mathcal {G}_\infty ^\perp $$

where $\mathcal {G}_\infty ={\text {Im}}\mathscr {G}$ is the space of infinitesimal gauge transformations and $\mathcal {G}_\infty ^\perp =\ker \mathscr {G}^\dagger$ is its $L^2$ orthogonal complement.

#### Proposition 3.3

Assume $k_+\ne 0$ or $k_-\ne 0$. Then, the operators $\mathscr {J}$ and $\mathscr {J}^G=\mathscr {J}+\mathscr {G}\mathscr {G}^{\dagger }$ have the same $L^2$-eigenvalues, corresponding to eigenmodes which are $L^2$-integrable.

#### Proof

We first observe that 0 is an eigenvalue of both $\mathscr {J}$ and $\mathscr {J}^G$ since any tangent vector to $\textsf{M}$ at $[(\boldsymbol{\phi },A)]$ is in the kernel of both $\mathscr {B}$ and $\mathscr {B}^G$. It remains to show that $\mathscr {J},\mathscr {J}^G$ have the same non-zero eigenvalues.

First assume $\boldsymbol{\xi }\ne 0$ is $L^2$ and $\mathscr {J}\boldsymbol{\xi }=\lambda ^2\boldsymbol{\xi }$ with $\lambda ^2\ne 0$. Then, it follows from (3.19) that $\mathscr {G}^\dagger \boldsymbol{\xi }=0$ and hence $\mathscr {J}^G\boldsymbol{\xi }=\mathscr {J}\boldsymbol{\xi }=\lambda ^2\boldsymbol{\xi }$. Hence, $\boldsymbol{\xi }$ is an $L^2$ eigenmode of $\mathscr {J}^G$ with the same eigenvalue.

Conversely, assume that $\boldsymbol{\xi }\ne 0$ is $L^2$ and $\mathscr {J}^G\boldsymbol{\xi }=\lambda ^2\boldsymbol{\xi }$ with $\lambda ^2\ne 0$. Denote by

$$ \boldsymbol{\xi }=\boldsymbol{\xi }^g+\boldsymbol{\xi }^{g\perp }, $$

where $\boldsymbol{\xi }^g\in \mathcal {G}_{\infty }$, $\boldsymbol{\xi }^{g\perp }\in \mathcal {G}_{\infty }^{\perp }$ its decomposition into $\mathcal {G}_\infty \oplus \mathcal {G}_\infty ^\perp$. Then we have, by (3.19),

$$ \mathscr {J}^G\boldsymbol{\xi }=\mathscr {J}\boldsymbol{\xi }^{g\perp }+\mathscr {G}\mathscr {G}^{\dagger }\boldsymbol{\xi }^g=\lambda ^2(\boldsymbol{\xi }^g+\boldsymbol{\xi }^{g\perp }), $$

whose components in $\mathcal {G}_\infty ^\perp$ and $\mathcal {G}_\infty$ are

3.34 $$\begin{aligned} \mathscr {G}\mathscr {G}^{\dagger }\boldsymbol{\xi }^g=\lambda ^2\boldsymbol{\xi }^g \end{aligned}$$

3.35 $$\begin{aligned} \mathscr {J}\boldsymbol{\xi }^{g\perp }=\lambda ^2\boldsymbol{\xi }^{g\perp } \end{aligned}$$

respectively. Hence, if $\boldsymbol{\xi }^{g\perp }\ne 0$, it is an $L^2$ eigenmode of $\mathscr {J}$, and the claim is established.

Finally, consider the case that $\boldsymbol{\xi }^{g\perp }=0$, so $\boldsymbol{\xi }=\boldsymbol{\xi }^g=\mathscr {G}(\chi )$ for some $\chi \in C^\infty (\Sigma )$. Then $\mathscr {G}^\dagger \mathscr {S}_1\boldsymbol{\xi }^g=\mathscr {G}^\dagger \mathscr {S}_1\mathscr {G}(\chi )=0$ by Eq. (3.27), so

$$ \mathscr {J}\mathscr {S}_1\boldsymbol{\xi }^g=\mathscr {J}^G S_1\boldsymbol{\xi }^g=\mathscr {S}_1\mathscr {J}^G\boldsymbol{\xi }^g=\lambda ^2\mathscr {S}_1\boldsymbol{\xi }^g, $$

by Lemma 3.1. $\mathscr {S}_1$ is an $L^2$ isometry, so $\mathscr {S}_1\boldsymbol{\xi }^g$ is an $L^2$ eigenmode of $\mathscr {J}$ with eigenvalue $\lambda ^2$, which completes the proof. □

Our next existence result builds on Theorem 3.2.

#### Theorem 3.4

Let $(\boldsymbol{\phi }, A)$ be a solution to the Bogomol’nyi equations on $\Sigma =\mathbb {R}^2$ with nonempty pair of divisors $(D_+,D_-)$. Then there exists a bound state of the BPS $\mathbb {C}P^1$ vortex if any of the following holds

- $$k_-=|D_-|=0$$ and $$\tau \in (0,1)$$
- $$k_+=|D_+|=0$$ and $$\tau \in (-1,0)$$
- τ=0.

#### Proof

In Theorem 3.2, we proved that if ψ is an eigenmode of $\mathscr {G}^{\dagger }\mathscr {G}$, then $\mathscr {S}_1\mathscr {G}\psi$ is an eigenmode of $\mathscr {J}$. Hence, if we can find a bound state of the Schrödinger operator (3.33), then $\mathscr {S}_1\mathscr {G}\psi$ represents a bound state of the vortex solution.

We can rewrite the eigenvalue problem for (3.33) as

3.36 $$\begin{aligned} -\nabla ^2\psi +V\psi =E\psi , \end{aligned}$$

where $E=\lambda ^2-(1-\tau ^2)$ and $V=\left| \boldsymbol{n}\times \boldsymbol{\phi }\right| ^2-(1-\tau ^2)=\tau ^2-(\boldsymbol{n}\cdot \boldsymbol{\phi })^2$. We observe that this new potential has the property that

$$ \lim _{|\boldsymbol{x}|\rightarrow \infty }V(\boldsymbol{x})=\lim _{|\boldsymbol{x}|\rightarrow \infty }[\tau ^2-(\boldsymbol{n}\cdot \boldsymbol{\phi })^2]=0, $$

due to the boundary conditions on $\boldsymbol{\phi }$. We now use a maximum principle to prove that $V(\boldsymbol{x})\le 0$ everywhere on the plane in the cases specified.

In order to do so, we first project stereographically from the South pole of $S^2$. This allows us to instead work with the complex field $u=\dfrac{\phi _1+i\phi _2}{1+\phi _3}$. The Bogomol’nyi equations then transform to

$$ \tilde{D}_1u=-i\tilde{D}_2u $$

$$ *dA=-\left( \tau -\dfrac{1-|u|^2}{1+|u|^2}\right) , $$

where now the covariant derivative is $\tilde{D}u=du-iAu$. Note we can write

3.37 $$\begin{aligned} |u|^2=\dfrac{1-\phi _3}{1+\phi _3}, \end{aligned}$$

and define

3.38 $$\begin{aligned} v=\ln {\dfrac{1-\phi _3}{1+\phi _3}}+\ln {\dfrac{1+\tau }{1-\tau }}. \end{aligned}$$

Using the boundary conditions on $\boldsymbol{\phi }$, we have $\lim _{|\boldsymbol{x}|\rightarrow \infty }v(\boldsymbol{x})=0$. Note that $D_+,D_-$ are the collections of points in $\mathbb {R}^2$ at which $\boldsymbol{\phi }=\boldsymbol{n}$ and $-\boldsymbol{n}$ respectively, so at each $\boldsymbol{p}\in D_+$, $v(\boldsymbol{p})=-\infty$ and at each $\boldsymbol{q}\in D_-$, $v(\boldsymbol{q})=\infty$.

Combining the two Bogomol’nyi equations, we obtain the so called Taubes equation

3.39 $$\begin{aligned} \nabla ^2v=2\dfrac{e^v-1}{\dfrac{1}{1-\tau }+\dfrac{e^v}{1+\tau }}+4\pi \sum _{\boldsymbol{q}\in D_-}\delta (\boldsymbol{q})-4\pi \sum _{\boldsymbol{p}\in D_+}\delta (\boldsymbol{p}). \end{aligned}$$

In [33], this equation was derived for the case τ=1. In [15], it was proven that (3.39), as a particular example of a more general Taubes-type equation considered by the author, admits a unique solution for any parameter $\tau \in (-1,1)$, that this solution is smooth away from $D_\pm$ and exponentially localized.

We observe that $\phi _3\ge \tau$ is equivalent to v≤0. We now assume $k_-=0$, so v only has negative singularities, and $\tau \in (0,1)$. If v is not everywhere non-positive, then since $\lim _{|\boldsymbol{x}|\rightarrow \infty }v(\boldsymbol{x})=0$, it must achieve a maximum at some $z_0$, such that $v(z_0)>0$. At this maximum, the Hessian ${{\,\textrm{Hess}\,}}(v(z_0))$ is negative semidefinite, hence $Tr({{\,\textrm{Hess}\,}}(v(z_0))\le 0$, but the trace of the Hessian is equal $\nabla ^2 v$, and therefore $\nabla ^2v(z_0)\le 0$. Observe that the LHS of (3.39) is non-positive at $z_0$, while the RHS is positive, leading to a contradiction. We deduce that $\phi _3(\boldsymbol{x})\ge \tau$ everywhere on the plane. Since τ>0, then we also have $(\phi _3)^2\ge \tau ^2$, and hence $V(\boldsymbol{x})\le 0$ everywhere on the plane.

The case $k_+=0$ and $\tau \in (-1,0)$ follows from a similar argument, where now the signs are flipped and we obtain $\phi _3\le \tau$ everywhere on the plane. Since τ<0, we reach the same conclusion that $(\phi _3)^2\ge \tau ^2$, and thus again $V(\boldsymbol{x})\le 0$.

The last case follows from the simple observation that τ=0 implies immediately that the potential $V(\boldsymbol{x})=\tau ^2-(\phi _3)^2$ is everywhere non-positive without any assumption on the number of vortices and anti-vortices.

A simple variational method with a cleverly chosen test function shows that any smooth potential $V(\boldsymbol{x})$ in two dimensions obeying $V(\boldsymbol{x})\le 0$ for all $\boldsymbol{x}\in \mathbb {R}^2$, $V(\boldsymbol{x})<0$ at some $\boldsymbol{x}$ and $\lim _{|\boldsymbol{x}|\rightarrow \infty }V(\boldsymbol{x})=0$ supports at least one bound state [32]. We note that for a bound state we must have E<0, so the eigenvalue is restricted to $\lambda ^2<1-\tau ^2$. We therefore conclude that there exists at least one shape mode of the vortex solution for each of the three cases considered. □

We can improve Theorem 3.4 by extending the range of τ for which at least one bound state exists by using an explicit test function and a continuity argument.

#### Theorem 3.5

Let $(\boldsymbol{\phi }, A)$ be a solution of the Bogomol’nyi equations on $\Sigma =\mathbb {R}^2$ with a fixed nonempty pair of divisors $(D_+,D_-)$. Then there exists a bound state of the BPS $\mathbb {C}P^1$ vortex if any of the following holds

- $$k_-=|D_-|=0$$ and $$\tau \in (-\tau _*,1)$$
- $$k_+=|D_+|=0$$ and $$\tau \in (-1,\tau _*)$$
- $$\tau \in (-\tau _*,\tau _*)$$,

where $\tau _*\in (0,1)$ depends on the divisor pair $(D_+,D_-)$.

#### Proof

In order to extend the range of τ, we need to construct a test function and apply the variational method.

Taking inner products on both sides and integrating by parts in (3.36) we can express the energy as the Rayleigh-Ritz quotient

3.40 $$\begin{aligned} E=\dfrac{\left\langle \psi ,H\psi \right\rangle _{L^2}}{\left\langle \psi ,\psi \right\rangle _{L^2}}=\dfrac{1}{||\psi ||^2_{L^2}}\int _{\mathbb {R}^2} \left( |d\psi |^2+V(r,\theta )\psi ^2\right) rdrd\theta , \end{aligned}$$

where the Hamiltonian operator is given by $H=L-(1-\tau ^2)=-\nabla ^2+V(r,\theta )$, and (r,θ) are polar coordinates on the plane.

Let $E_0$ be the ground state energy of H. The variational method states that the energy of an arbitrary state is always at least $E_0$. Hence, if we can find a state for which the Rayleigh quotient is negative, then we can infer that the true ground state energy $E_0$ is also negative, which leads to the existence of at least one bound state.

Given τ, consider the family of test functions

3.41 $$\begin{aligned} \psi _{\alpha ,\tau }(r)=e^{-\sqrt{1-\tau ^2-\alpha ^2}r}, \end{aligned}$$

with $\alpha ^2<1-\tau ^2$. Plugging into (3.40) we obtain the associated energy

$$ E(\alpha ,\tau )=\dfrac{2}{\pi }(1-\tau ^2-\alpha ^2)\int _{\mathbb {R}^2} \left( 1-\alpha ^2-(\phi _3)^2\right) \psi _{\alpha ,\tau }^2rdrd\theta , $$

where recall that the potential can be written as $V=\tau ^2-(\phi _3)^2$. Let

3.42 $$\begin{aligned} I_{\alpha }=\int _{\mathbb {R}^2} \left( 1-\alpha ^2-(\phi _3)^2\right) \psi _{\alpha ,\tau }^2rdrd\theta , \end{aligned}$$

and note that $I_\alpha$ is finite for all $\alpha ^2<1-\tau ^2$, since $\lim _{|\boldsymbol{x}|\rightarrow \infty }\phi _3(\boldsymbol{x})=\tau$ and $\psi _{\alpha ,\tau }$ is exponentially decaying. Furthermore, if $\alpha ^2=1-\tau ^2$, then

$$ I_{\sqrt{1-\tau ^2}}=\int _{\mathbb {R}^2} \left( \tau ^2-(\phi _3)^2\right) rdrd\theta $$

is also finite due to the exponential convergence of $\phi _3$ to τ.

Before continuing our argument for extending the range of τ for which shape modes exist, we first need to establish that $E(\alpha ,\tau )$ is continuous in τ in a small neighborhood of τ=0. Notice that the energy depends on $\phi _3$ which itself depends on τ, and hence, we need to establish some form of continuity of $\phi _3$ with respect to τ. The following theorem, whose proof is presented in an appendix, suffices:

#### Theorem 3.6

Given a disjoint pair of divisors ($D_+,D_-$), let $(A^\tau ,\boldsymbol{\phi }^\tau )$ be the solution (unique up to gauge) of the Bogomol’nyi Eqs. (2.4) and (2.5) with parameter τ. Then there exists $\epsilon \in (0,1)$ such that the map

$$\begin{aligned}\Phi :(-\epsilon ,\epsilon )&\rightarrow L^2(\mathbb {R}^2)\\ \tau&\mapsto \phi _3^\tau -\tau \end{aligned}$$

is $$C^1$$.

In the remaining part of the proof we will refer to $\phi _3$ by $\phi _3^\tau$ to emphasize its dependence on τ.

A simple calculation shows that

$$ E(\alpha , \tau )=1-\alpha ^2-\dfrac{2}{\pi }(1-\tau ^2-\alpha ^2)\int _{\mathbb {R}^2} (\phi _3^\tau )^2\psi _{\alpha ,\tau }^2rdrd\theta . $$

It therefore remains to prove that $\tau \mapsto \phi _3^\tau \psi _{\alpha ,\tau }$ is continuous in $L^2$. Recalling $\tau \mapsto \Phi (\tau )=\phi _3^\tau -\tau$ is continuous on τ in a small neighborhood of τ=0 by Theorem 3.6, we can rewrite

3.43 $$\begin{aligned} \int _{\mathbb {R}^2}(\phi _3^\tau )^2\psi _{\alpha ,\tau }^2d^2x= &   \int _{\mathbb {R}^2} (\phi _3^\tau -\tau )^2\psi _{\alpha ,\tau }^2d^2x+2\tau \int _{\mathbb {R}^2} \phi _3^\tau \psi _{\alpha ,\tau }^2d^2x-\tau ^2\int _{\mathbb {R}^2} \psi _{\alpha ,\tau }^2d^2x \nonumber \\= &   \int _{\mathbb {R}^2} \Phi (\tau )^2\psi _{\alpha ,\tau }^2d^2x+2\tau \int _{\mathbb {R}^2} \Phi (\tau )\psi _{\alpha ,\tau }^2d^2x+\tau ^2\int _{\mathbb {R}^2} \psi _{\alpha ,\tau }^2d^2x \end{aligned}$$

Note

$$ \tau ^2\int _{\mathbb {R}^2} \psi _{\alpha ,\tau }^2d^2x=\dfrac{\tau ^2\pi }{2(1-\tau ^2-\alpha ^2)} $$

is obviously continuous in τ in a small neighborhood of τ=0. Now we deal with the remaining two integrals. Consider $\tau ,\tau _0\in (-\epsilon ,\epsilon )$, where $0<\epsilon \ll 1$. Then we have

3.44 $$\begin{aligned}  &   \left| \int _{\mathbb {R}^2}\Phi (\tau )\psi _{\alpha ,\tau }^2d^2x -\int _{\mathbb {R}^2}\Phi (\tau _0)\psi _{\alpha ,\tau _0}^2d^2x\right| \nonumber \\= &   \left| \int _{\mathbb {R}^2} (\Phi (\tau )-\Phi (\tau _0))\psi _{\alpha ,\tau }^2d^2x+\int _{\mathbb {R}^2} \Phi (\tau _0)(\psi _{\alpha ,\tau }^2-\psi _{\alpha ,\tau _0}^2)d^2x\right| \nonumber \\\le &   \left| \langle \Phi (\tau )-\Phi (\tau _0),\psi _{\alpha ,\tau }^2\rangle _{L^2}\right| +\left| \langle \psi _{\alpha ,\tau }-\psi _{\alpha ,\tau _0},\Phi (\tau _0)(\psi _{\alpha ,\tau }+\psi _{\alpha ,\tau _0})\rangle _{L^2}\right| \nonumber \\\le &   \Vert \Phi (\tau )-\Phi (\tau _0)\Vert _{L^2}\Vert \psi _{\alpha ,\tau }^2\Vert _{L^2}+\Vert \psi _{\alpha ,\tau }-\psi _{\alpha ,\tau _0}\Vert _{L^2}\Vert \Phi (\tau _0)(\psi _{\alpha ,\tau }+\psi _{\alpha ,\tau _0})\Vert _{L^2}\nonumber \\\rightarrow &   0 {\text { as }}\tau \rightarrow \tau _0 \end{aligned}$$

since Φ and $\tau \mapsto \psi _{\alpha ,\tau }$ are $L^2-$continuous in a small neighourhood of τ=0, and the other $L^2-$norms are finite due to their integrands’ exponential decay.

In a very similar manner, we also prove that $\tau \mapsto \int _{\mathbb {R}^2} \Phi (\tau )^2\psi _{\alpha ,\tau }^2d^2x$ is continuous in a small neighborhood around τ=0. This completes the proof that $\tau \mapsto \int _{\mathbb {R}^2}(\phi _3^\tau )^2\psi _{\alpha ,\tau }^2$ is continuous, and therefore we deduce that $E(\alpha ,\tau )$ is continuous on τ in a small neighborhood around τ=0.

We can now use this fact to prove the statement of the theorem. Assume τ=0 and $k_\pm$ are not both 0. Then obviously $I_1<0$. Since $I_{\alpha }$ depends continuously on α (the α dependence of the integrand is explicit through polynomial and exponential functions), there exists $\alpha _{*}<1$ such that $I_{\alpha _{*}}<0$, and hence $E(\alpha _{*},0)<0$. Since $E(\alpha _{*},\tau )$ is continuous in τ, we deduce there exists a small neighborhood $\tau \in (-\tau _*,\tau _*)$ for which $E(\alpha _{*},\tau )<0$, and hence, a bound state exists. Notice this argument depends on the solution $(\boldsymbol{\phi },A)$, and therefore $\tau _*$ depends on the locations of the (anti-)vortices, information which is encoded in the divisor pair $(D_+,D_-)$.

We can now easily extend this argument to the cases where $k_-=0$ or $k_+=0$. The above argument proved there exists a bound state for any $k_\pm$ if $\tau \in (-\tau _*,\tau _*)$, so in particular we can set $k_-=0$. From Theorem (3.4), a bound state exists for $k_-=0$ and $\tau \in (0,1)$. Combining these two results we deduce there exists a bound state when $k_-=0$ and $\tau \in (-\tau _*,1)$. Similarly, we deduce we have a bound state when $k_+=0$ and $\tau \in (-1,\tau _*)$. □

## Radially symmetric vortices

In this section, we apply our previous results in the particular case where we assume radial symmetry and all vortices are concentrated at the origin. The Bogomol’nyi equations reduce to two coupled ODEs. We compute the shape modes numerically by solving the Bogomol’nyi equations and the ODE reduced from (3.36) together as a system.

### Symmetry reduction on $\Sigma =\mathbb {R}^2$.

If we assume radial symmetry, the locations of all (anti)-vortices are constrained to the origin of $\mathbb {R}^2$. Therefore, we can only have either N North vortices ($k_+=N$, $k_-=0$) or N South anti-vortices ($k_+=0$, $k_-=N$). Choosing the former case, as the latter follows in a very similar manner, we look for solutions using the hedgehog ansatz

4.1 $$\begin{aligned} \boldsymbol{\phi }(x)=(\sin {f(r)}\cos {N\theta }, \sin {f(r)}\sin {N\theta }, \cos {f(r)}) \end{aligned}$$

4.2 $$\begin{aligned} A=Na(r)d\theta , \end{aligned}$$

where (r,θ) are polar coordinates on the plane.

Plugging these in the Bogomol’nyi equations, they reduce to a system of first-order ODEs for a(r) and f(r):

4.3 $$\begin{aligned} f'= \dfrac{N}{r}(1-a)\sin {f} \end{aligned}$$

4.4 $$\begin{aligned} a'=-\dfrac{r}{N}(\tau -\cos {f}). \end{aligned}$$

The condition $\boldsymbol{\phi }(0)=(0,0,1)$ that all vortices are superposed at the origin implies that f(0)=0. Furthermore, regularity of the gauge field at the origin imposes a(0)=0, and the boundary conditions we set before reduce to

$$ \lim _{r\rightarrow \infty }{a'(r)}=0 {\text {, }} \lim _{r\rightarrow \infty }a(r)=1 {\text {, }}\lim _{r\rightarrow \infty }{\cos {f(r)}}=\tau .$$

Hence, as $r\rightarrow \infty$, $\boldsymbol{\phi }$ approaches the vacuum manifold given by

$$ \left( \sqrt{1-\tau ^2}\cos {N\theta }, \sqrt{1-\tau ^2}\sin {N\theta }, \tau \right) . $$

### Asymptotic behavior and magnetic flux

By looking at the asymptotic behavior of (4.3) and (4.4) in the two regimes r→0, $r\rightarrow \infty$, we can find analytic approximations for the functions f(r), a(r).

- As r→0, we find
  4.5 $$\begin{aligned} a(r)\approx \dfrac{r^2}{2N}(1-\tau ) \end{aligned}$$
  4.6 $$\begin{aligned} f(r)\approx Ar^N, \end{aligned}$$
  where A is an arbitrary constant and will be used as a shooting constant to find the vortex solution numerically.
- As $r\rightarrow \infty$, we find
  4.7 $$\begin{aligned} a(r)\approx 1-\dfrac{q}{2N\pi }rK_1\left( r\sqrt{1-\tau ^2}\right) \end{aligned}$$
  4.8 $$\begin{aligned} f(r)\approx \arccos {\tau }-\dfrac{q}{2\pi }K_0\left( r\sqrt{1-\tau ^2}\right) , \end{aligned}$$
  where $K_0$, $K_1$ are modified Bessel functions of the second kind. The constant q represents the strength of the asymptotic vortex charge in the particle intepretation of Speight [26, 27]. These asymptotics hold in the case $\tau \in (-1,1)$.

A quick calculation using the hedgehog ansatz shows that $F=Na'(r)dr\wedge d\theta$. Hence, we can directly compute the total magnetic flux

$$ \int _{\mathbb {R}^2}F=2\pi N, $$

which is quantized since N is an integer. One should note that this result depends crucially on our choice to restrict τ to (-1,1). If $\tau =\pm 1$, the magnetic flux is not quantized, but it is equal to Nα, where $\alpha =\lim _{r\rightarrow \infty }{a(r)}$ [24].

### Shape modes of a cylindrically symmetric $\mathbb {C}{\text {P}}^1$N-vortex

We follow the strategy given by Theorem 3.2 which allows us to easily compute at least one shape mode of the vortex solution by first solving for the eigenfunctions of the Schr$\ddot{\text {o}}$dinger operator (3.33). To prove that this strategy indeed gives us all the possible shape modes in the case of a radially symmetric N-vortex, we then construct the operator $\mathscr {B}^{G}\mathscr {B}^{G\dagger }$ in radial coordinates, and show it reduces to the single PDE given by the eigenvalue problem of (3.33).

In the case of radial symmetry, substituting the hedgehog ansatz (4.1) shows that the operator (3.33) becomes

$$ L=\Delta + \sin ^2f(r). $$

To find the eigenfunctions of L, we need to solve the eigenvalue problem $L\Psi =\lambda ^2\Psi$. Motivated by the U(1) symmetry, we make the ansatz $\Psi (r,\theta )=\psi (r)e^{ik\theta }$, where k is an integer. The eigenvalue problem then reduces to

4.9 $$\begin{aligned} -\psi ''-\dfrac{1}{r}\psi '+\left( \dfrac{k^2}{r^2}+\sin ^2{f}\right) \psi =\lambda ^2\psi , \end{aligned}$$

Since the potential depends explicitly on the radial profile of the Higgs field, we need to solve (4.9) together with (4.3) and (4.4). Theorem 3.2 showed that the shape mode is then given by

$$ \mathscr {S}_1\mathscr {G} \Psi (r)=\begin{pmatrix} (\boldsymbol{n}-(\boldsymbol{n}\cdot \boldsymbol{\phi })\boldsymbol{\phi })\psi (r)e^{ik\theta }\\ r\psi '(r)e^{ik\theta }d\theta -i\dfrac{k}{r}\psi e^{ik\theta }dr \end{pmatrix}=\begin{pmatrix} \boldsymbol{\eta }\\ \alpha \end{pmatrix}. $$

and can be visualized in Figs. 2, 3 and 4 for the cases N=1,2,3, and k=0, where we define

4.10 $$\begin{aligned} \psi _1(r):=|\eta _3|=(1-(\phi _3)^2)\psi (r) \end{aligned}$$

4.11 $$\begin{aligned} \psi _2(r):= r\psi '(r). \end{aligned}$$

### Shape mode asymptotics

By looking at the asymptotic behaviour of the Schr$\ddot{\text {o}}$dinger Eq. (4.9) in the two regimes r→0 and $r\rightarrow \infty$, we can find analytic approximations for the bound state solutions ψ(r).

- As r→0, we find
  4.12 $$\begin{aligned} \psi (r)\approx C_0 J_k(\lambda r) \end{aligned}$$
  where $J_k$ is a Bessel function of the first kind and $C_0$ is an arbitrary constant. The ODE is linear, and hence, $C_0$ can be chosen arbitrarily. In particular, we can always rescale it such that $C_0=1$, which we will use in the numerical calculations.
- As $r\rightarrow \infty$, we find
  4.13 $$\begin{aligned} \psi (r)\approx CK_k\left( \sqrt{1-\tau ^2-\lambda ^2}r\right) \end{aligned}$$
  where $K_k$ is a modified Bessel function of the second kind and C is a constant. These asymptotics hold in the case $\tau \in (-1,1)$. Notice we obtain bound states if and only if $\lambda ^2<1-\tau ^2$.

### Numerical results

We solve numerically for the shape modes in the radially symmetric case on $\mathbb {R}^2$, where the vortices are all located at the origin. Since the eigenvalue problem for the Jacobi operator involves the Higgs and gauge fields, we must couple it to the Bogomol’nyi equations and solve them together as a system. To the best of our knowledge, none of these equations are integrable and we must resort to numerical methods.

**Fig. 1:**
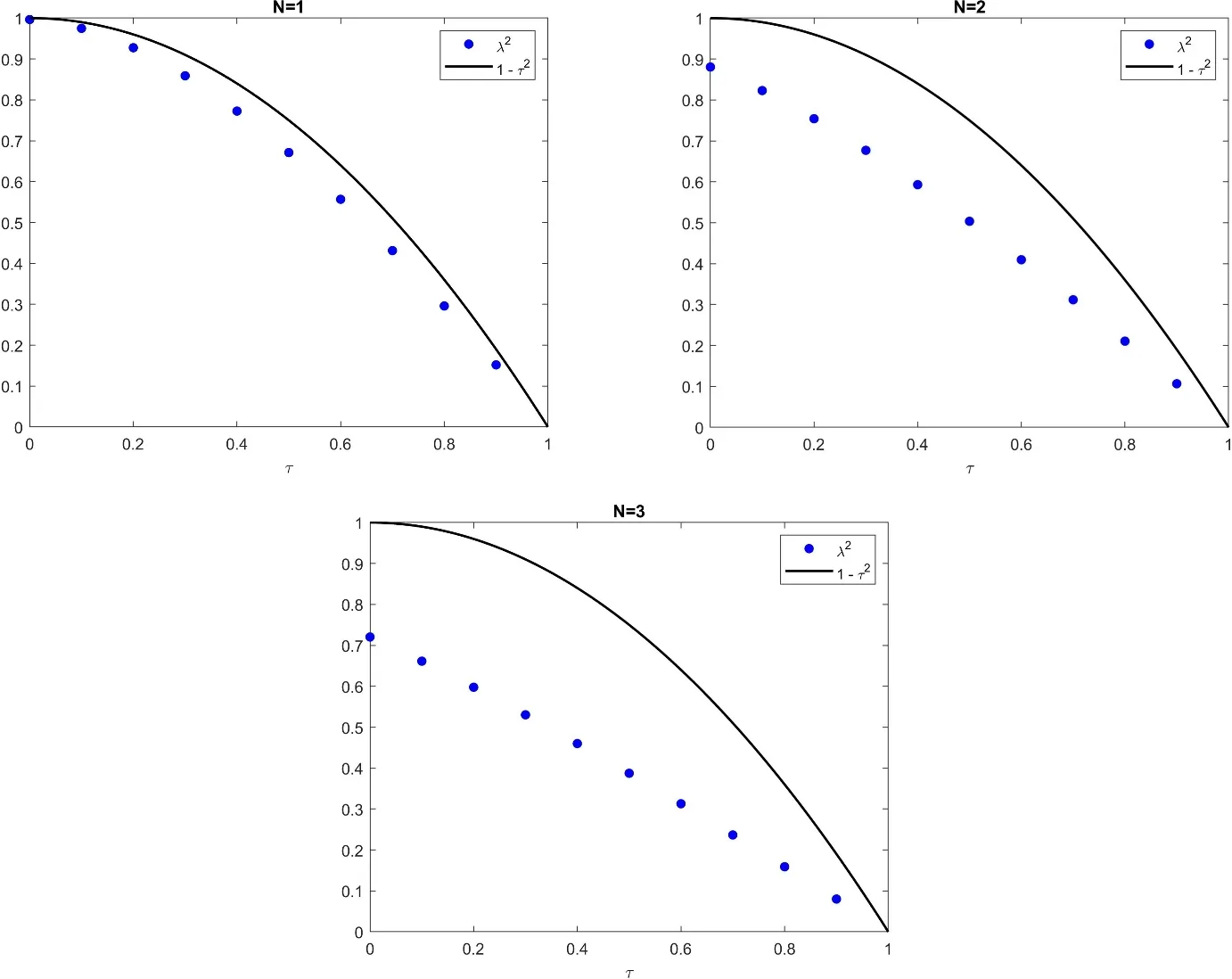
The eigenvalue $\lambda ^2$ of the Jacobi operator vs τ for a North vortex solution with N=1, N=2 and N=3. In each case, the system was solved for k=0. The black curve represents the scattering threshold $1-\tau ^2$

To solve the Bogomol’nyi equations coupled to the Schr$$\ddot{\text {o}}$$dinger equation for ψ(r), we first build two solutions, one starting from the left, with initial conditions given by the asymptotics for small r in (4.5), (4.6), (4.12), and one starting from the right, with initial conditions given by the asymptotics for large r in (4.7), (4.8), (4.13). We integrate the left solution over the range $$[r_0,r_1]$$, and the right solution over $$[r_1, r_{\text {max}}]$$. We usually take $$r_0=10^{-6}$$, $$r_1=2$$, and $$r_{max}=20$$. Recall the asymptotics depend on the constants A, q, C and λ which can be treated as shooting parameters. To match the left and right solutions at the point $$r=r_1$$, we find the shooting constants A, q, C for which the left and right profiles for f(r), a(r) and $\psi^{\prime }\left(r\right)$ agree within $$10^{-7}$$ error using the Newton–Raphson method, and simultaneously apply a bisection method to find the value of λ for which the left and right profiles for ψ(r) agree within the same error. A separate bisection method is required to find the eigenvalue due to the fact that for N=1 it is always very close to the scattering threshold (see first plot of Fig. 1), and therefore incorporating it in the Newton–Raphson numerical scheme can lead to non-convergence.

The system of ODEs is solved using the MATLAB function ode45, which is based on a Runge–Kutta (4,5) method.

**Fig. 2:**
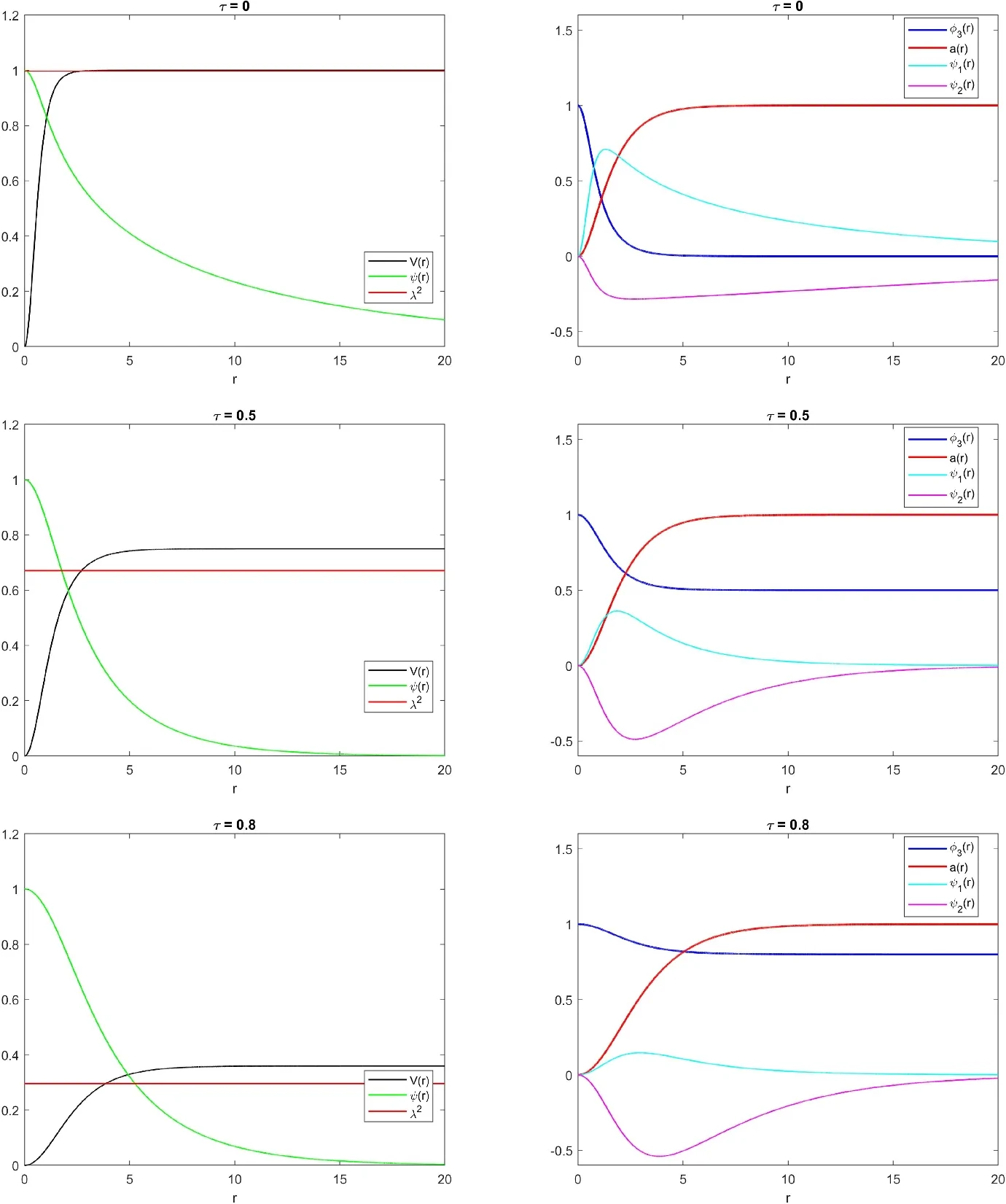
On the left column we present the wavefunction ψ(r) and the potential of the Schr$\ddot{\text {o}}$dinger Eq. (4.15), along with the eigenvalue $\lambda ^2$. On the right column we present the radial profiles of the gauge field a(r) and the gauge invariant quantity $\phi _3=\cos {f(r)}$, along with the shape mode perturbations $\psi _1(r)$ and $\psi _2(r)$ given by $\mathscr {S}_1\mathscr {G} \Psi (r)$ (see (4.10) and (4.11) for their explicit definition). All quantities were computed for a North vortex solution with N=1, k=0, and different values of τ, chosen to be 0, 0.5 and 0.8. The eigenvalues were computed numerically to be $\lambda ^2\approx 0.99654$, $\lambda ^2\approx 0.67116$, $\lambda ^2\approx 0.29596$, respectively

**Fig. 3:**
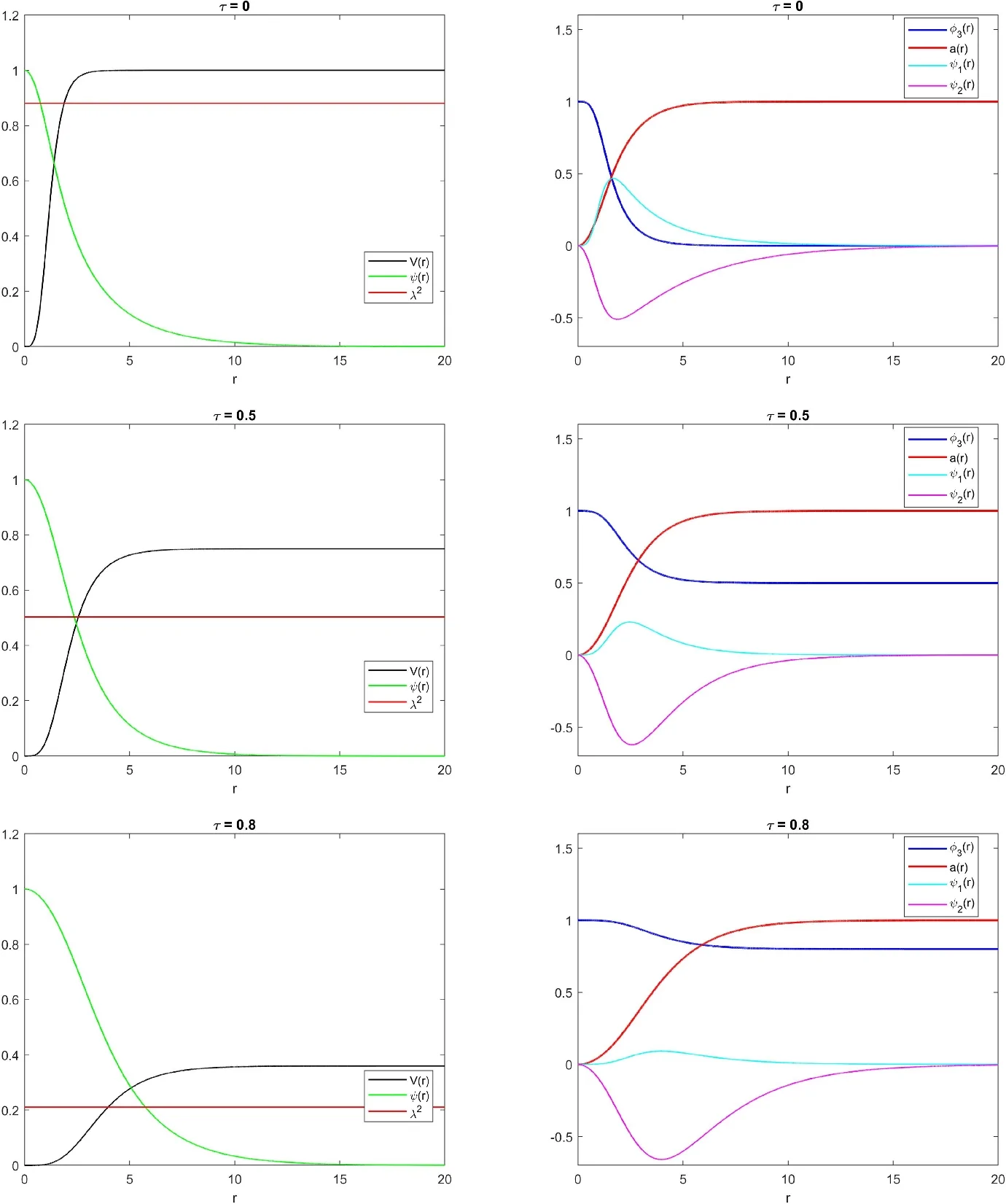
On the left column we present the wavefunction ψ(r) and the potential of the Schr$\ddot{\text {o}}$dinger Eq. (4.15), along with the eigenvalue $\lambda ^2$. On the right column we present the radial profiles of the gauge field a(r) and the gauge invariant quantity $\phi _3=\cos {f(r)}$, along with the shape mode perturbations $\psi _1(r)$ and $\psi _2(r)$ given by $\mathscr {S}_1\mathscr {G} \Psi (r)$ (see (4.10) and (4.11) for their explicit definition). All quantities were computed for a North vortex solution with N=2, k=0, and different values of τ, chosen to be 0, 0.5 and 0.8. The eigenvalues were computed numerically to be $\lambda ^2\approx 0.88054$, $\lambda ^2\approx 0.50355$, $\lambda ^2\approx 0.21058$, respectively

**Fig. 4:**
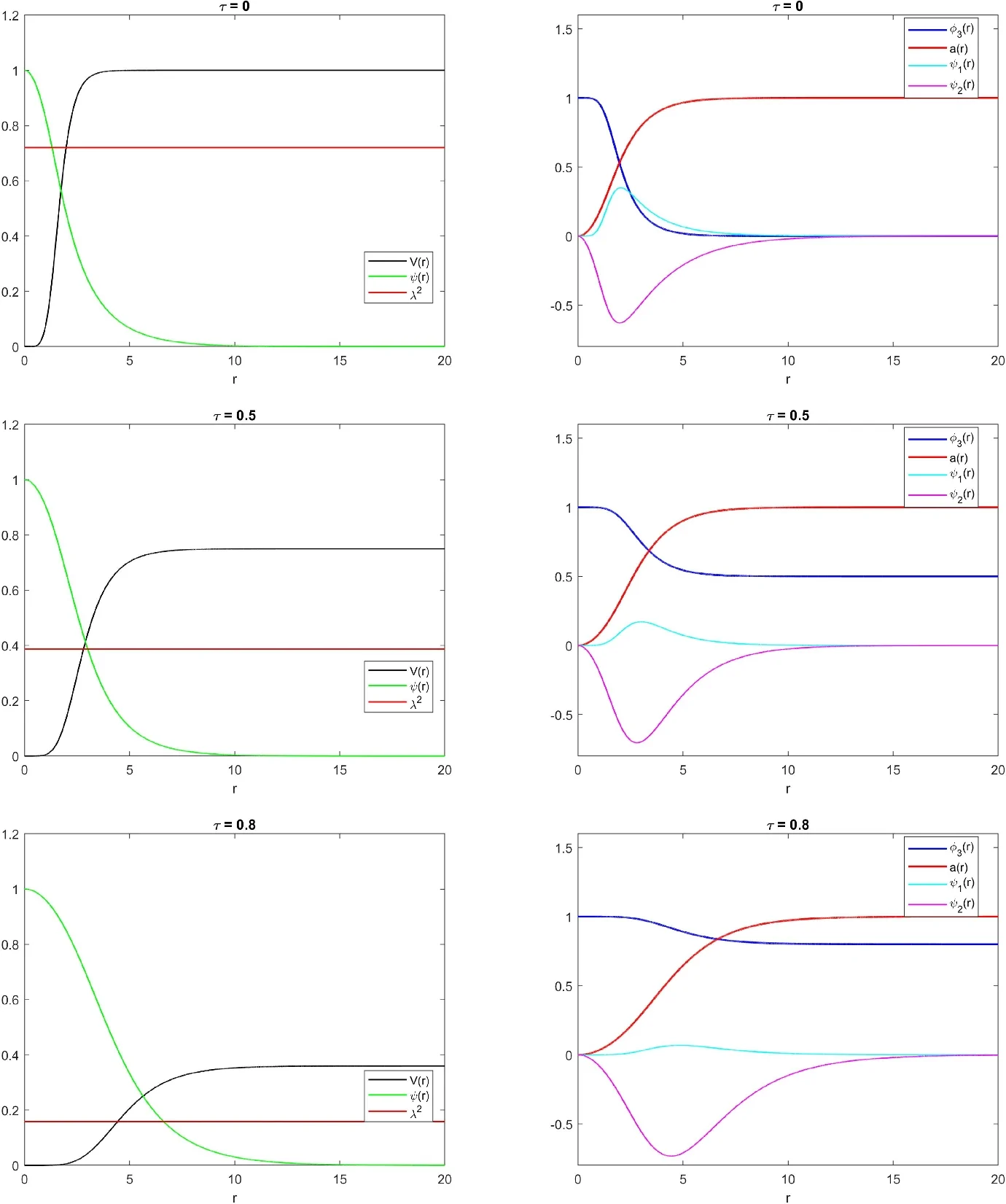
On the left column, we present the wavefunction ψ(r) and the potential of the Schr$\ddot{\text {o}}$dinger Eq. (4.15), along with the eigenvalue $\lambda ^2$. On the right column, we present the radial profiles of the gauge field a(r) and the gauge invariant quantity $\phi _3=\cos {f(r)}$, along with the shape mode perturbations $\psi _1(r)$ and $\psi _2(r)$ given by $\mathscr {S}_1\mathscr {G} \Psi (r)$, see (4.10) and (4.11) for their explicit definition. All quantities were computed for a North vortex solution with N=3, k=0, and different values of τ, chosen to be 0, 0.5 and 0.8. The eigenvalues were computed numerically to be $\lambda ^2\approx 0.72042$, $\lambda ^2\approx 0.38743$, $\lambda ^2\approx 0.15896$

For the case N=1, the Schr$\ddot{\text {o}}$dinger Eq. (4.15) admits bound states only for k=0. Higher integer values for k result in potentials which are always above the bound states threshold $1-\tau ^2$. In Fig. 1, we show the eigenvalue $\lambda ^2$ of the Jacobi operator for various values of τ in the interval [0,1), for solutions with vortex numbers N=1,2,3. Figures 2, 3 and 4 show the eigenfunction ψ(r), the Schr$\ddot{\text {o}}$dinger potential, and the shape mode perturbations $\psi _1(r)$, $\psi _2(r)$ for the cases N=1,2,3 and specific values of τ.

In Theorem 3.4, we proved that for $k_-=0$ and $\tau \in [0,1)$ then there exists at least one shape mode of the vortex solution. This is indeed confirmed by the numerical results presented in Fig. 1. In Theorem 3.5, we further remarked that since the energy depends continuously on τ, there must be some $\tau _*>0$ such that we can extend the range of τ for which a shape mode exists to $\tau \in (-\tau _*,1)$. Our numerical strategy shows that, in the case of radial vortices with N=1, this threshold is $\tau _*\approx 0.001$.

A surprising result is that for a single vortex the eigenvalue $\lambda ^2$ is always very close to the scattering threshold $1-\tau ^2$, see Fig. 1. Hence, the binding energy of the shape mode of a 1-vortex for all values of τ is very low in magnitude. Even for mid-range values of τ, for which the gap between the eigenvalue and the threshold appears to increase (see Figs. 1 and 2), the binding energy is, in fact, still small. For instance, in the case τ=0.5, the binding energy is E≈-0.0788. This does not happen, for example, in the case of the Abelian-Higgs model [6], where for N=1 the shape mode has eigenvalue ≈0.777476 and the bound state threshold is 1, giving E≈0.222524. This suggests that weakly bound shape modes could be a feature of the $\mathbb {C}P^1$ model.

**Fig. 5:**
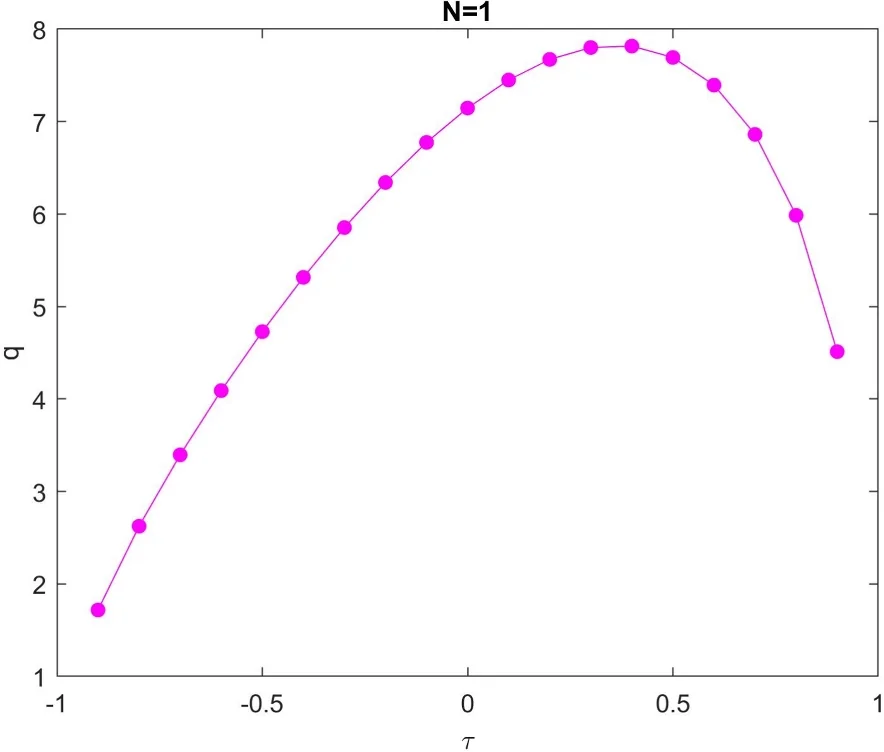
The asymptotic vortex charge q vs τ for the case N=1

In Fig. 5, we compute the asymptotic charge q for a 1-vortex given in (4.7) and (4.8) against various values of $\tau \in [0,1)$. This charge appears in the computation of the conformal factor of the $L^2-$metric of the vortex-antivortex moduli space in the large separation regime, as shown in [23] for the case τ=0.

### Are there any other shape modes?

An important question is whether the eigenvalue problem (4.9) gives all the possible shape modes for a radially symmetric N-vortex. To answer this, we note that, if $\boldsymbol{\xi }$ is an eigensection of $\mathscr {J}$ with eigenvalue $\lambda ^2>0$, it is an eigensection of $\mathscr {J}^G=\mathscr {B}^{G\dagger }\mathscr {B}^G$ with the same eigenvalue, and $\mathscr {B}^G\boldsymbol{\xi }$ (which is not 0) is an eigensection of $\mathscr {B}^G\mathscr {B}^{G\dagger }$ with the same eigenvalue. Hence, it suffices to compute the spectrum of the operator $\mathscr {B}^G\mathscr {B}^{G\dagger }$ acting on $\mathscr {B}^G(\mathcal {PERT})\subset \mathcal {BOG}$.

We first note that if $\tilde{\boldsymbol{\xi }}=\mathscr {B}^G(\boldsymbol{\xi })$, then

$$ \tilde{\boldsymbol{\xi }}=\begin{pmatrix} \dfrac{1}{\sqrt{2}}\tilde{h}(\boldsymbol{e_\theta }\otimes dr-\boldsymbol{e_r}\otimes rd\theta )+\dfrac{1}{\sqrt{2}}\tilde{g}(\boldsymbol{e_r}\otimes dr+\boldsymbol{e_\theta }\otimes rd\theta )\\ \tilde{\beta } \\ \tilde{\gamma } \end{pmatrix}, $$

where dr, rdθ is an orthonormal basis of $T^{*}\Sigma$,

$$ \boldsymbol{e_r}=(\cos {f}\cos {N\theta },\cos {f}\sin {N\theta },-\sin {f}) $$

$$ \boldsymbol{e_\theta }=(\sin {N\theta },-\cos {N\theta },0) $$

is an orthonormal basis of $\boldsymbol{\phi }^*TS^2$, and $\tilde{h}$, $\tilde{g}$, $\tilde{\beta }$, $\tilde{\gamma }$ are functions of r,θ. Then

$$ \mathscr {B}^{G\dagger }\tilde{\boldsymbol{\xi }}=\begin{pmatrix} -\left( \partial _r \tilde{h}+\dfrac{1}{r}\partial _\theta \tilde{g}+\dfrac{1}{r}\left( 1+N(1-a)\cos {f}\right) \tilde{h}+\tilde{\gamma } \sin {f}\right) \boldsymbol{e_\theta }-\\ -\left( \partial _r \tilde{g}-\dfrac{1}{r}\partial _\theta \tilde{h}+\dfrac{1}{r}\left( 1+N(1-a)\cos {f}\right) \tilde{g}-\tilde{\beta } \sin {f}\right) \boldsymbol{e_r}\\ \left( \partial _r \tilde{\gamma }+\dfrac{1}{r}\partial _\theta \tilde{\beta }+\tilde{h}\sin {f} \right) dr+ \left( -\partial _r \tilde{\beta }+\dfrac{1}{r}\partial _\theta \tilde{\gamma }+\tilde{g}\sin {f}\right) rd\theta \end{pmatrix}. $$

Finally, we can compute $\mathscr {B}^G\mathscr {B}^{G\dagger }\tilde{\boldsymbol{\xi }}=\lambda ^2\tilde{\boldsymbol{\xi }}$ to obtain a system of decoupled PDEs:

$$ -\nabla ^2\,h+\dfrac{2}{r^2}\left( 1+N(1-a)\cos {f}\right) \partial _{\theta }g+\left( \dfrac{1}{r^2}\left( 1+N(1-a)\cos {f}\right) ^2+1-\tau \cos {f}\right. $$

$$\left. +\dfrac{N^2}{r^2}(1-a)^2\sin ^2{f}\right) h=\lambda ^2\,h $$

$$ -\nabla ^2\,g-\dfrac{2}{r^2}\left( 1+N(1-a)\cos {f}\right) \partial _{\theta }h+\left( \dfrac{1}{r^2}\left( 1+N(1-a)\cos {f}\right) ^2+1-\tau \cos {f}\right. $$

$$\left. +\dfrac{N^2}{r^2}(1-a)^2\sin ^2{f}\right) g=\lambda ^2\,g $$

$$ -\nabla ^2\beta +\beta \sin ^2{f}=\lambda ^2\beta $$

$$ -\nabla ^2\gamma +\gamma \sin ^2{f}=\lambda ^2\gamma , $$

where we have dropped the tilde symbol to avoid cumbersome notation.

Since the functions $h,g,\gamma ,\beta$ are periodic in θ, we can decompose them into Fourier modes

$$ h(r,\theta )=\Theta _{Nk}(r)\cos {k\theta }, g(r,\theta )=\Theta _{Nk}(r)\sin {k\theta } $$

$$ \beta (r,\theta )=\psi _{Nk}(r)\cos {k\theta }, \gamma (r,\theta )=\psi _{Nk}(r)\sin {k\theta }, $$

where k is an integer, labeling each Fourier sector. Notice the other half of the Fourier space can be obtained by applying the symmetry $\mathscr {S}_2$ explained in Lemma 3.1. The PDEs now reduce to a decoupled pair of second-order ODEs for each *k*:

4.14 $$\begin{aligned} -\Theta ''-\dfrac{1}{r}\Theta '+\left( \dfrac{1}{r^2}\left( k+1+N(1-a)\cos {f}\right) ^2+1-\tau \cos {f}+\dfrac{N^2}{r^2}(1-a)^2\sin ^2{f}\right) \Theta =\lambda ^2\Theta \end{aligned}$$

4.15 $$\begin{aligned} -\psi ''-\dfrac{1}{r}\psi '+\left( \dfrac{k^2}{r^2}+\sin ^2{f}\right) \psi =\lambda ^2\psi , \end{aligned}$$

where we have dropped the subscripts for ψ and Θ. These are both 2D radial Schr$\ddot{\text {o}}$dinger equations in a central potential depending on the radial profiles of the Higgs and gauge fields, f(r) and a(r), respectively.

We immediately notice that the Schr$\ddot{\text {o}}$dinger ODE (4.15) is in fact the radial reduction of eigenvalue problem of the operator (3.33) examined in Theorem 3.2, which we derived at the beginning of the chapter, in (4.9). Numerical investigation shows that the potential corresponding to the Schr$\ddot{\text {o}}$dinger Eq. (4.14) is always above the mass threshold, implying the only bound state solutions are given by solving Eq. (4.15). Hence, we deduce that, in the particular case of radial symmetry, the eigenfunctions of the operator examined in Theorem 3.2 give all the possible shape modes of a radially symmetric N-vortex.

## Conclusions

In this paper, we investigated the internal modes of a general vortex solution in the gauged $\mathbb {C}P^1$ sigma model with target $S^2$. We developed a formalism for computing the Jacobi operator obtained from the second variation of the energy functional which exploits the Bogomol’nyi decomposition. This operator can be factorized into a product of two first-order operators, one of which is the $L^2-$adjoint of the other. To ensure the gauge is fixed, we built a new Jacobi operator which proved to have the same spectrum as the original Jacobi operator. This new operator allows for a much easier computation of the shape modes. In particular, for a general vortex solution on $\mathbb {R}^2$, we proved the existence of at least one shape mode for τ close to 0. We also proved existence of at least one shape mode for all pure North vortices (having empty South divisor $D_-$) for $\tau \in (0,1)$. The construction of these shape modes requires one only to solve a single scalar PDE, the eigenvalue problem of a certain rather simple Schr$\ddot{\text {o}}$dinger operator. This is very surprising, as the computation of shape modes generally requires solving the eigenvalue problem for the Jacobi operator itself, a system of coupled PDEs. We also remarked that this formalism consists of a general approach, and can therefore be applied to other models which admit a Bogomol’nyi decomposition.

We have used a numerical scheme based on the Newton–Raphson method to obtain the shape mode and its corresponding eigenvalue for a North vortex with topological charge N=1,2,3 and for various values of τ. Our numerics agreed with the results proven analytically in Sect. 3 and further revealed that the shape mode for a 1-vortex in this model is generally weakly bound for all values of $\tau \in (0,1)$.

We remark that, as a by-product of this numerical scheme, we have computed the asymptotic parameter q(τ) for a single North vortex which, as far as we are aware, has not previously been reported in the literature (except for τ=0 [23]). This quantity, which we plot in Fig. 5, may be endowed with physical significance via the point vortex formalism [26]. The asymptotic fields of the vortex coincide with the solution of the linearization of the model about the vacuum in the presence of a scalar monopole of charge -q(τ) and a magnetic dipole of moment $-q(\tau )\hat{\textbf{k}}$ at the vortex core (the origin). By an obvious symmetry, the South antivortex corresponds to a point particle with monopole charge q(-τ) and dipole moment $q(-\tau )\hat{\textbf{k}}$. By analyzing the forces between widely separated and slowly moving point particles of this type within the linearized field theory, one can derive a conjectural asymptotic formula for the $L^2$ metric on the moduli space of static $(k_+,k_-)$ vortices, valid in the region where all (anti)vortices are well separated. The result, due to Garcia Lara [14], is

$$ g\sim 2\pi \sum _{r}(1-\sigma _r\tau )|dz_r|^2 -\frac{1}{4\pi }\sum _{r}\sum _{s\ne r}\sigma _r\sigma _sq(\sigma _r\tau )q(\sigma _s\tau )K_0\left( \sqrt{1-\tau ^2}|z_r-z_s|\right) |dz_r-dz_s|^2, $$

where $z_1,z_2,\ldots ,z_{k_++k_-}\in \mathbb {C}$ are the positions of the (anti)vortices and $\sigma _r=+1$ for a North vortex and -1 for a South antivortex. Having computed q(τ), we now know these formulae quantitatively.

A generalized Abelian-Higgs theory has recently been developed in [31] which allows for a Bogomol’nyi decomposition and the coexistence of vortices and anti-vortices for an energy functional with a general potential. It would be interesting to explore the existence of shape modes in these models using the formalism presented in this paper. As pointed out in [31], one can choose a suitable potential to control the curvature of strings in a cosmological setting. Computation of shape modes in such a scenario could therefore offer much information about the dynamics of cosmic strings in the early universe.

Another natural question is whether some version of this formalism can be modified to construct shape modes of Chern-Simons deformed vortices, either in the conventional model with target $\mathbb {C}$ [20] or the model with target $\mathbb{C}\mathbb{P}^1$ [17]. These models still have a Bogomol’nyi decomposition, but it is conceptually more subtle: The solutions are stationary rather than static (they have kinetic energy), and one of the “Bogomol’nyi" equations is Gauss’s law, the Euler-Lagrange equation associated with $A_0$. So this extension, if possible at all, is likely to be quite nontrivial.

It would also be interesting to study the shape modes of vortices in a model which admits some form of integrability. An example would be the Abelian-Higgs model (with target space $\mathbb {C}$) on the hyperbolic plane of curvature -1/2 [30]. A shape mode of the charge one vortex has recently been found explicitly in this model [28], with eigenvalue $\lambda ^2=1$. One should note that this is below the scattering threshold, which is 9/8 rather than 1 due to curvature effects. Another setting in which integrability comes into play is when $\Sigma =\mathbb {R}^2$ is endowed with a conformal factor leading to an integrable Taubes equation, as observed in [13] for N coincident vortices. Such properties may allow for a more analytical description of the shape modes, which is not reliant on numerical methods.

## Data Availability

Data generated during the current study are available from the corresponding author on reasonable request.

## Appendix A. Proof of Theorem 3.6

We will use the Implicit Function Theorem (IFT) to prove the continuity of the solution in τ. We first establish the setting by defining a perturbation function which is regular and satisfies its own PDE. Then we define the function to which we will apply the IFT. Before we do this, we first need to check the requirements of the IFT hold true in our context. Hence, our proof is divided into multiple steps intended to check each of the requirements.

For a fixed (and disjoint) divisor pair $(D_+,D_-)$ denote by $(\boldsymbol{\phi }^\tau ,A^\tau )$ the solution of (2.4), (2.5) with $\boldsymbol{\phi }^{-1}(\pm \boldsymbol{n})=D_\pm$. This is known to exist, to be unique up to gauge, to be smooth and, in an appropriate sense, be exponentially spatially localized [15]. Consider the stereographic projection from the South pole of $S^2$

$$ u_\tau :=\dfrac{\phi _1^\tau +i\phi _2^\tau }{1+\phi _3^\tau }. $$

From this, we easily see

A.1 $$\begin{aligned} |u_\tau |=\sqrt{\dfrac{1-\phi _3^\tau }{1+\phi _3^\tau }}\rightarrow \sqrt{\dfrac{1-\tau }{1+\tau }} {\text { as }}|\boldsymbol{x}|\rightarrow \infty . \end{aligned}$$

Since $u_\tau$ has the same collections of zeros $D_+$ and poles $D_-$ for all τ, we may obtain $u_\tau$ as a deformation of $u_0$. That is, there exists $h_\tau :\mathbb {R}^2\rightarrow \mathbb {R}$ such that

A.2 $$\begin{aligned} u_\tau =\sqrt{\frac{1-\tau }{1+\tau }}e^{h_\tau /2}u_0. \end{aligned}$$

Note that $h_\tau (\infty )=0$ by (A.1). This field satisfies the first Bogomol’nyi equation if and only if [19] we deform the connexion $A^0$ as

$$ A^\tau =A^0-\frac{1}{2}*dh_\tau , $$

a connexion whose curvature is

$$ *F_\tau =*F_0+\frac{1}{2}\Delta h_\tau =\phi _3^0+\frac{1}{2}\Delta h_\tau . $$

Hence, the deformed pair $(A^\tau ,u_\tau )$ satisfies both Bogomol’nyi equations if and only if

A.3 $$\begin{aligned} \Delta h_\tau +2\left( \phi _3^0-\Psi (\tau ,h_\tau )\right) =0, \end{aligned}$$

where

A.4 $$\begin{aligned} \Psi (\tau ,h)=\frac{(1+\tau )(1+\phi _3^0)-(1-\tau )(1-\phi _3^0)e^{h}}{(1+\tau )(1+\phi _3^0)+(1-\tau )(1-\phi _3^0)e^{h}}-\tau \end{aligned}$$

has been defined so that $\phi _3^\tau -\tau =\Psi (\tau ,h_\tau )$. We are going to use the fact that $h_\tau$ satisfies the above PDE to show that $\phi _3^\tau$ depends continuously on τ. Note that $h_0=0$ by construction.

### Claim 1

Ψ is a $$C^1$$ map $$(-1,1)\times H^2(\mathbb {R}^2)\rightarrow L^2(\mathbb {R}^2)$$.

### Proof

By the Sobolev embedding theorem [2], $H^{2}(\mathbb {R}^2)\subset C^0_B(\mathbb {R}^2)$, where $C^0_B(\mathbb {R}^2)$ is the space of all continuous bounded functions on $\mathbb {R}^2$ which is a Banach space with norm $||f||_{C^0}=\sup _{\boldsymbol{x}\in \mathbb {R}^2}|f(\boldsymbol{x})|$, and the inclusion map is continuous. Hence, there exists C>0 such that, for all $h\in H^{2}$ we have $\Vert h\Vert _{C^0}\le C\Vert h\Vert _{H^2}$.

Furthermore, we know $H^2(\mathbb {R}^2)$ is a Banach algebra [2]. That is, there exists C>0 such that, for all $h,g\in H^2$, $hg\in H^2$, and $\Vert hg\Vert _{H^2}\le C\Vert h\Vert _{H^2}\Vert g\Vert _{H^2}$.

Note also that $Q:h\mapsto e^h-1$ is a smooth map $H^2\rightarrow H^2$. To see this, note that it is defined by the power series

$$ Q(h)=\sum _{k=1}^\infty \frac{h^k}{k!} $$

which is absolutely convergent on the Banach algebra $H^2$.

We can simplify Ψ as follows

$$ \Psi (\tau ,h)=-\frac{(1-\tau ^2)[(e^h-1)-\phi _3^0(e^h+1)]}{(1+\tau )(1+\phi _3^0)+(1-\tau )(1-\phi _3^0)e^{h}}=:\frac{K(h,\tau )}{L(h,\tau )}, $$

and partition the plane $P_+=(\phi _3^0)^{-1}([0,\infty ))$, $P_-=(\phi _3^0)^{-1}(-\infty ,0))$. Note that on $P_+$

$$ L>(1+\tau )\ge 1-|\tau | $$

and on $$P_-$$

$$ L>(1-\tau )e^{-\Vert h\Vert _{C^0}}\ge (1-|\tau |)e^{-C\Vert h\Vert _{H^2}} $$

by the Sobolev embedding theorem. Hence, on the whole plane

$$ |\Psi (\tau ,h)|\le (1+|\tau |)e^{C\Vert h\Vert _{H^2}}\left[ |Q(h)|+(e^{C\Vert h\Vert _{H^2}}+1)|\phi _3^0|\right] . $$

It follows that $\Psi (\tau ,h)^2$ is integrable, since $Q(h)\in H^2\subset L^2$ and $\phi _3^0$ is smooth and exponentially localized.

Hence, Ψ maps $(-1,1)\times L^2 \rightarrow H^2$ as claimed. That Ψ is $C^1$ follows from elementary estimates on the difference quotient. □

Consider the map

$$ G:(-1,1)\times H^2(\mathbb {R}^2)\rightarrow L^2(\mathbb {R}^2) $$

$$ G(\tau ,h)=\Delta h+2(\phi _3^0-\Psi (\tau ,h)) $$

so that the PDE defining $h_\tau$ is $G(\tau ,h_\tau )=0$. $\Delta :H^2\rightarrow L^2$ is a bounded linear map, so it follows immediately from Claim 1 that *G* is a $C^1$ map. Define

$$ \bar{G}:H^2(\mathbb {R}^2)\rightarrow L^2(\mathbb {R}^2) $$

$$ \bar{G}(h)=G(0,h). $$

Then a simple calculation shows

$$ d\bar{G}|_{h=0}=\Delta +1-(\phi _3^0)^2. $$

We will denote this operator $\mathscr {L}:H^2\rightarrow L^2$ because it coincides precisely with the Schrödinger operator introduced in Theorem 3.2, in the case τ=0.

### Claim 2

The operator $\mathscr {L}$ is bounded and injective.

Boundedness follows trivially from the fact that $\phi _3^0$ is smooth and bounded. To prove injectivity, we start from $\mathscr {L}f=0$, multiplying by f and integrating by parts we obtain

$$ \int _{\mathbb {R}^2}|df|^2d^2x+\int _{\mathbb {R}^2}(1-(\phi _3^0)^2)f^2d^2x=0. $$

Since $V=1-(\phi _3^0)^2\ge 0$ and vanishes only on the finite set $D_+$, we deduce f=0. Therefore $\mathscr {L}$ is injective. □

We next wish to prove that $\mathscr {L}$ is, in fact, invertible. So we need to show that $\mathscr {L}$ is surjective and has bounded inverse. To do this, we will need to show that a certain class of multiplication operators $H^2\rightarrow L^2$ are compact. Recall that this means that the operator maps every bounded subset of $H^2$ to a subset of $L^2$ with compact closure.

### Claim 3

Let $U:\mathbb {R}^2\rightarrow \mathbb {R}$ be a continuous function such that $U=O(|\boldsymbol{x}|^{-2-\epsilon })$ as $|\boldsymbol{x}|\rightarrow \infty$ for some ϵ>0. Then the map $H^2(\mathbb {R}^2)\rightarrow L^2(\mathbb {R}^2)$, f↦Uf is compact.

### Proof

We use weighted Sobolev spaces. For a review, see [21]. Let

$$\begin{aligned} \rho :\mathbb {R}^2\rightarrow \mathbb {R},\quad \rho (\boldsymbol{x})=\sqrt{1+|\boldsymbol{x}|^2}. \end{aligned}$$

For any $\beta \in \mathbb {R}$, let $L^2$, $H^2$, $L^2_{0,\beta }$, $L^2_{2,\beta }$ be the completions of $C^\infty _c(\mathbb {R}^2)$ (the space of smooth functions of compact support) with respect to the following norms:

$$\begin{aligned} \Vert f\Vert _{L^2}^2&= \int _{\mathbb {R}^2}f^2\,d^2x\\ \Vert f\Vert _{H^2}^2&= \int _{\mathbb {R}^2}\big (f^2+|\nabla f|^2+|\nabla \nabla f|^2\big )\,d^2x\\ \Vert f\Vert _{L^2_{0,\beta }}^2&= \int _{\mathbb {R}^2}\big (\rho ^{-\beta }f\big )^2\,\frac{d^2x}{\rho ^2}\\ \Vert f\Vert _{L^2_{2,\beta }}^2&= \int _{\mathbb {R}^2}\big ((\rho ^{-\beta }f)^2+|\rho ^{1-\beta }\nabla f|^2+|\rho ^{2-\beta }\nabla \nabla f|^2\big )\,\frac{d^2x}{\rho ^2}, \end{aligned}$$

in which $\nabla \nabla f$ denotes the Hessian matrix of *f*. Theorem 4.18 of [21] tells us that when $\beta <\delta$ the embedding $L^2_{2,\beta }\rightarrow L^2_{0,\delta }$ is compact.

The embedding

$$\begin{aligned} H^2\rightarrow L^2_{2,1} \end{aligned}$$

is continuous because $\rho ^p\le 1$ for p≤0. The embedding

$$\begin{aligned} L^2_{2,1} \rightarrow L^2_{0,1+\epsilon } \end{aligned}$$

is compact by Theorem 4.18 of [21]. Our assumptions on *U* mean that $U\le C\rho ^{-2-\epsilon }$ for some C>0. So the map

$$\begin{aligned} L^2_{0,1+\epsilon }\rightarrow L^2_{0,-1}=L^2,\quad f\mapsto Uf \end{aligned}$$

is continuous. So the map $H^2\rightarrow L^2$, f↦Uf is a composition of a compact map with two bounded linear maps. Therefore it is compact. □

### Claim 4

The operator $\mathscr {L}$ is Fredholm of index zero.

### Proof

Since $\phi _3^0$ is exponentially decaying as $|\boldsymbol{x}|\rightarrow \infty$, we can apply Claim 3 to $U=-(\phi _3^0)^2$. Then the map $P:H^2(\mathbb {R}^2)\rightarrow L^2(\mathbb {R}^2)$, $f\mapsto -(\phi _3^0)^2f$ is compact.

The operator $\mathscr {L}_0:H^2(\mathbb {R}^2)\rightarrow L^2(\mathbb {R}^2)$, $\mathscr {L}_0=\Delta +1$ is clearly injective. Surjectivity follows via Fourier transforms. Therefore it is Fredholm of index zero.

So $\mathscr {L}=\mathscr {L}_0+P$ is equal to the sum of a Fredholm operator $\mathscr {L}_0$ and a compact operator P; hence, it is also Fredholm [11] with index

$$ ind({\mathscr {L})}=ind(\mathscr {L}_0)=0. $$

□

### Claim 5

The operator $\mathscr {L}$ is bijective, with bounded inverse.

### Proof

Claims 2 and 4 combined show that $\mathscr {L}$ is bijective. Hence, we know that $\mathscr {L}$ is a continuous (bounded) linear operator between two Banach spaces which is also bijective. It is known by Corollary 2.7 from [12] that the inverse of such an operator is bounded. □

### Claim 6

There exists ϵ>0 such that the map

$$\begin{aligned} (-\epsilon , \epsilon )&\rightarrow H^2(\mathbb {R}^2)\\ \tau&\rightarrow h_\tau , \end{aligned}$$

corresponding to the solution of the PDE (A.3), is a $C^1$ map.

### Proof

We have just established that $\mathscr {L}=d\bar{G}_0:H^2\rightarrow L^2$ is invertible, so the claim follows from the implicit function theorem applied to *G* at (τ,h)=(0,0). □

### Claim 7

The map

$$\begin{aligned}\Phi :(-\epsilon ,\epsilon )&\rightarrow L^2(\mathbb {R}^2)\\ \tau&\rightarrow \Psi (\tau ,h_\tau )=\phi _3^\tau -\tau \end{aligned}$$

is $$C^1$$.

Since $\tau \mapsto h_\tau$ is $C^1$, and $(\tau ,h)\mapsto \Psi (\tau , h)$ given in (A.4) is $C^1$, the composition $\tau \mapsto \Psi (\tau ,h_\tau )=\phi _3^\tau -\tau$ is also $C^1$. This concludes the proof of the theorem. □

## References

[1] Abrikosov, A.A.: On the Magnetic properties of superconductors of the second group. Sov. Phys. JETP **5**, 1174–1182 (1957)

[2] Adams, R.A., Fournier, J.J.F.: Sobolev Spaces, vol. 140, 2nd edn. Press, Acad (2003)

[3] Alonso-Izquierdo, A., Bachmaier, M., Wereszczynski, A.: From BPS geodesics to mode-driven dynamics in the scattering of multiple BPS vortices, (2026). arXiv:2603.04495

[4] Alonso-Izquierdo, A., Garcia Fuertes, W., Manton, N.S., Mateos Guilarte, J.: Spectral flow of vortex shape modes over the BPS 2-vortex moduli space. J. High Energ. Phys, (2024)

[5] Alonso-Izquierdo, A., Fuertes, W.G., Mateos Guilarte, J.: A note on BPS vortex bound states. Phys. Lett. B **753**, 29–33 (2016)

[6] Alonso-Izquierdo, A., Fuertes, W.G., Mateos Guilarte, J.: Dissecting zero modes and bound states on BPS vortices in Ginzburg-Landau superconductors. J. High Energ. Phys 74 (2016)

[7] Alonso-Izquierdo, A., Manton, N.S., Mateos Guilarte, J., Rees, M., Wereszczynski, A.: Dynamics of excited BPS three-vortices. Phys. Rev. D **111**, 105021 (2025)

[8] Alonso-Izquierdo, A., Manton, N.S., Mateos Guilarte, J., Wereszczynski, A.: Collective coordinate models for 2-vortex shape mode dynamics. Phys. Rev. D **110**, 085006 (2024)

[9] Alonso-Izquierdo, A., Mateos Guilarte, J., Rees, M., Wereszczynski, A.: Spectral wall in collisions of excited Abelian Higgs vortices. Phys. Rev. D **110**, 065004 (2024)

[10] Alonso-Izquierdo, A., Miguélez-Caballero, D., Queiruga, J.: Spectral structure of fluctuations around n-vortices in the Abelian-Higgs model, (2025). arXiv:2505.05039v2

[11] Brezis, H.: Compact operators. spectral decomposition of self-adjoint compact operators, pp. 157–179. Springer (2011)

[12] Brezis, H.: The uniform boundedness principle and the closed graph theorem, pp. 31–54. Springer (2011)

[13] Dunajski, M., Gavrea, N.: Elizabethan vortices. Nonlinearity **36**(8), 4169 (2023)

[14] Garcia-Lara, R.I.: Geometric models of soliton vortex dynamics, (2020). arXiv:2105.00597v1

[15] Han, J.: Existence of topological multivortex solutions in the self-dual gauge theories. Proc. R. Soc. Edinb., Sect. A, Math. **130**(6), 1293–1309 (2000)

[16] Karmakar, M., Menon, G.I., Ganesh, R.: Vortex-core order and field-driven supersolidity. Phys. Rev. B **96**, 174501 (2017)

[17] Kimm, K., Lee, K., Lee, T.: Anyonic Bogomol’nyi solitons in a gauged o(3) model. Phys. Rev. D **53**(8), 4436 (1996) 10.1103/physrevd.53.4436 10020443

[18] Krusch, S., Rees, M., Winyard, T.: Scattering of vortices with excited normal modes. Phys. Rev. D **110**, 056050 (2024)

[19] García Lara, R.I., Speight, J.M.: The Geometry of the Space of Vortices on a Two-Sphere in the Bradlow Limit. Commun. Math. Phys. **403**(3), 1411–1427 (2023)

[20] Lee, C., Lee, K., Min, H.: Self-dual Maxwell Chern–Simons solitons. Phys. Lett. B **252**(1), 79–83 (1990)

[21] Marshall, S.P.: Deformations of special lagrangian submanifolds, University of Oxford (2002). (Ph.D. thesis)

[22] Miguélez-Caballero, D., Navarro-Obregón, S., Wereszczynski, A.: Moduli space metric of the excited vortex. Phys. Rev. D **111**, 105008 (2025)

[23] Romão, N.M., Speight, J.M.: The geometry of the space of BPS vortex-antivortex pairs. Commun. Math. Phys. **379**, 723–772 (2020)

[24] Schroers, B.J.: Bogomol’nyi solitons in a gauged O(3) sigma model. Phys. Lett. B **356**, 291–296 (1995)

[25] Sibner, L., Sibner, R., Yang, Y.: Abelian Gauge theory on Riemann surfaces and new topological invariants. Proc. R. Soc. Lond. A **456**(1995), 593–613 (2000)

[26] Speight, J.M.: Static intervortex forces. Phys. Rev. D **55**, 3830–3835 (1997)

[27] Speight, J.M., Winyard, T.: Intervortex forces in competing-order superconductors. Phys. Rev. B **103**, 014514 (2021)

[28] Stuart, D.: (2026). (private communication)

[29] Vilenkin, A., Shellard, E.P.S.: Cosmic Strings and Other Topological Defects. Cambridge Univ, Press (1994)

[30] Witten, E.: Some exact multipseudoparticle solutions of classical Yang–Mills theory. Phys. Rev. Lett. **38**, 121–124 (1977)

[31] Xu, A., Yang, Y.: Bogomol’nyi equations and coexistence of vortices and antivortices in generalized Abelian-Higgs theories. Proc. R. Soc. A 481, (2025)

[32] Yang, K., de Llano, M.: Simple variational proof that any two-dimensional potential well supports at least one bound state. Am. J. Phys. **57**, 85–86 (1989)

[33] Yang, Y.: A necessary and sufficient condition for the existence of multisolitons in a self-dual gauged sigma model. Commun. Math. Phys. **181**, 485–506 (1996)

[34] Yang, Y.: Coexistence of vortices and antivortices in an abelian gauge theory. Phys. Rev. Lett. **80**, 26–29 (1998)

